# Past Trends and Future Directions of Cardiac Regenerative Medicine – A Systematic Analysis of Clinical Trial Registries

**DOI:** 10.1007/s12265-024-10563-1

**Published:** 2024-10-03

**Authors:** Maaike Wulfse, Mats T. Vervoorn, Jantijn J. G. J. Amelink, Elisa M. Ballan, Saskia C. A. de Jager, Joost P. G. Sluijter, Pieter A. Doevendans, Peter-Paul M. Zwetsloot, Niels P. Van der Kaaij

**Affiliations:** 1 Department of Cardiothoracic Surgery, Division of Heart & Lungs, University Medical Center Utrecht, P.O. Box 85500, Utrecht, 3508 GA The Netherlands; 2 Netherlands Heart Institute, Utrecht, The Netherlands; 3 Department of Cardiology, Laboratory of Experimental Cardiology, Division of Heart & Lungs, University Medical Center Utrecht, Utrecht, The Netherlands; 4 Circulatory Health Research Center, Regenerative Medicine Utrecht, University Utrecht, Utrecht, The Netherlands; 5 Department of Cardiology, Division of Heart & Lungs, University Medical Center Utrecht, Utrecht, The Netherlands

**Keywords:** Regenerative Medicine, Cell Therapy, Gene Therapy, Tissue Engineering, Cardiovascular Disease, Clinical Trials, Cardiology

## Abstract

Cell therapy, gene therapy, and tissue engineering have been explored as potential strategies to repair or regenerate damaged cardiac tissue. Despite the presence of encouraging preclinical data, clinical trials of regenerative cardiac therapies have yielded mixed results. Our study aimed to investigate the fate of all registered clinical trials within regenerative cardiac medicine, with the purpose of exploring the potential role of publication bias (or trial-completion bias), how published and unpublished research affects the field, and to draw lessons and recommendations for future clinical trials. In this analysis, we show that only a third of all registered trials has yielded results and that a significant number of trials are not completed. Furthermore, we identified significant heterogeneity in study design, study phase, funding, specific therapies used, primary outcome measures and methods of outcome assessment. These observations might hinder the successful translation of cardiac regenerative therapies into clinical practice.

**Graphical Abstract:**

Our study concludes that there is substantial underreporting of results from clinical trials within regenerative cardiac therapy. Coupled with significant heterogeneity in study design, this hinders progression of the field.

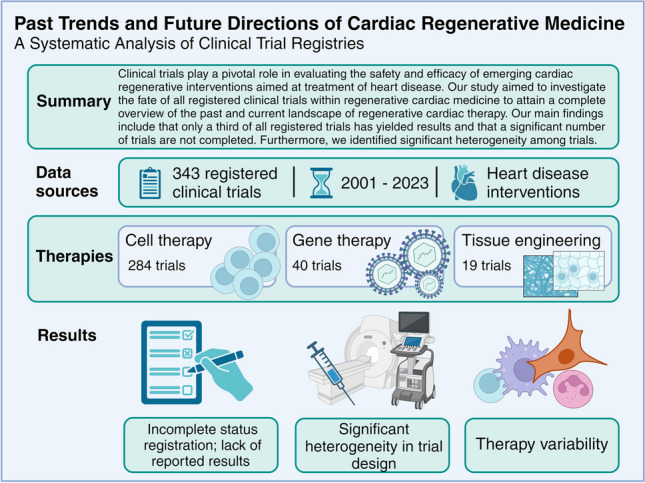

**Supplementary Information:**

The online version contains supplementary material available at 10.1007/s12265-024-10563-1.

## Introduction

Regenerative medicine is a rapidly emerging field that aims to restore the normal structure and function of human tissue through cell therapy, gene therapy and tissue engineering [1]. In short, cell therapy involves the transfer of viable cellular material, either autologous or allogeneic, into a patient to promote repair of tissues [2]. Gene therapy can be used to replace, repair or inactivate a specific gene, or to introduce specific genetic material to treat or prevent disease [3]. Tissue engineering involves implantation of a combination of living cells, biocompatible materials and/or suitable biochemical and physical factors to create tissue-like structures with the purpose of repairing or replacing the function of a failing organ [4]. Within cardiology, different regenerative therapies have been explored as potential strategies to cure heart disease by repairing or regenerating damaged cardiac tissue. This is especially relevant given the low regenerative capacity of the heart and the rising incidence of chronic conditions that affect heart function [5, 6].

Along the translational axis, clinical trials are the bridge between preclinical studies and widespread clinical adoption. They play a pivotal role in the advancement of regenerative cardiac therapy to clinical patient care. However, despite encouraging preclinical data, clinical trials that explored cell therapy, gene therapy or tissue engineering have yielded mixed results. This raises questions about the effectiveness of these therapeutic interventions [6–9]. Besides, the degree of publication bias that stems from unreported and incomplete clinical trials remains unexplored and may result in overestimation of therapeutic efficacy, or otherwise wrongful conclusions.

While review studies generally provide an overview of published clinical trial results, it remains unclear to what extent clinical trials into regenerative cardiac therapy remain unreported or incomplete. We therefore conducted a systematic analysis of clinical trial registries to provide a full overview of the past and current landscape of regenerative cardiac therapy and explore to what extend the field is shaped by both published and unpublished research. Furthermore, we draw lessons and formulate recommendations for future clinical trials based on the observations made and identified trends to improve the conduct of clinical trials within this field.

## Methods

### Search Strategy

A targeted search for clinical trials on regenerative cardiac therapy was performed on January 1st, 2024, within Clinicaltrials.gov, Cochrane library, the EU clinical trials register, and the WHO International Clinical Trials Registry Platform (ICTRP). Keywords included cell therapy, gene therapy, and tissue engineering, as well as heart disease and heart failure, explicated in Appendix 1.

### Study Selection

Three independent researchers (MW, MV, JA) screened the identified trials for inclusion. Trials were included if the studied intervention involved regenerative cardiac therapy (cell therapy, gene therapy, or tissue engineering) and assessed therapeutic safety or efficacy. Inconsistencies were resolved by critical discussion amongst the authors (MW, MV, JA). Trials were excluded if the study design was non-interventional or concerned technical aspects of the aforementioned therapies (such as bone marrow harvesting or vector construction).

Identified trials were downloaded from their registries and uploaded into Microsoft Office Excel 2016 for duplicate removal, screening, and data extraction.

### Data Extraction

The extracted data included: category of regenerative therapy (cell therapy, gene therapy, or tissue engineering), completion status, study phase, year of registration, availability of results (included in registry or via a linked journal article with corresponding trial registration number), source of funding, targeted heart disease and demographic data. Heart disease was classified into five main groups: heart failure (HF), myocardial infarction, coronary artery disease, congenital heart disease and arrhythmia. HF was further subdivided into ischemic HF, genetic HF, HF with preserved ejection fraction, other forms of HF, and non-specified HF. Funding was categorized into industry-affiliated and non-industry-affiliated based on whether an industrial partner was listed as primary sponsor or collaborator. Primary outcome and method of outcome assessment were noted for all trials, as well as delivery route and specific cells, genes, and tissue engineering techniques involved.

### Statistical Analysis

Results were extracted into data tables and descriptive statistics was applied. Statistical analysis was performed to compare differences among groups using the Chi-squared test for categorical variables. Statistical significance was set at a p-value of < 0.05. Analyses were performed using IBM SPSS (IBM Corp. Released 2019. IBM SPSS Statistics for Windows, Version 26.0. Armonk, NY: IBM Corp).

## Results

**Fig. 1:**
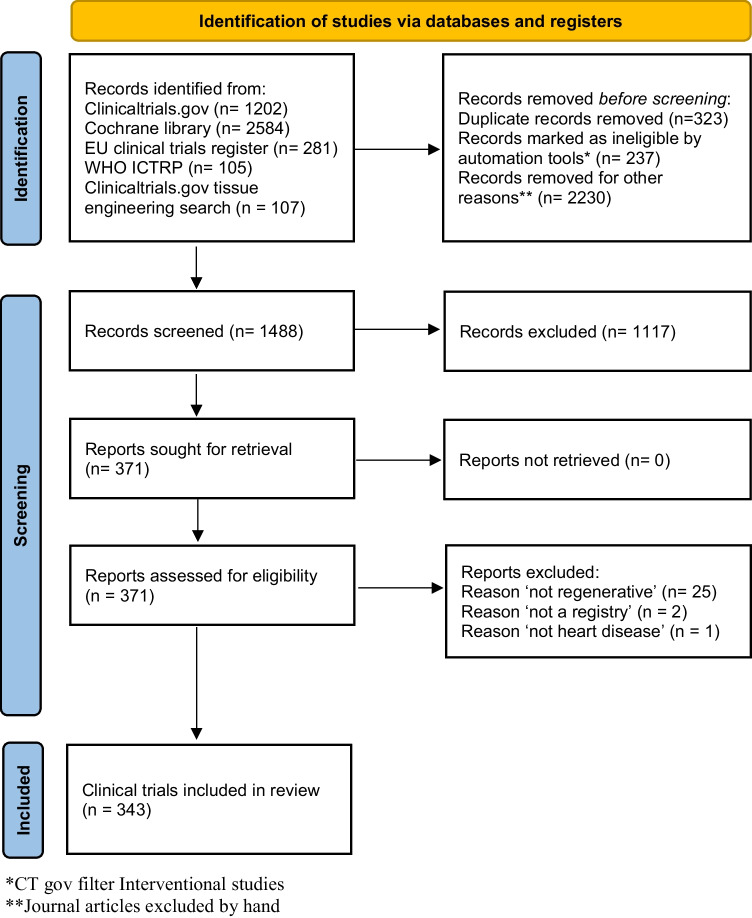
Search flowchart

The systematic search resulted in 4278 records, of which 1489 remained after duplicate removal. After screening, a total of 343 clinical trial registrations were included. Figure 1 shows the search flowchart. Results from all included registries are listed in Appendix 2.

### Trial Characteristics

Trial characteristics are summarized in Table 1. Among the 343 included registered clinical trials, 284 (82.8%) investigated cell therapy, 40 (11.7%) gene therapy, and 19 (5.5%) tissue engineering. The most targeted disease was heart failure (HF) (177 trials, 51.6%), followed by myocardial infarction (90, 26.2%), coronary artery disease (56, 16.3%), congenital heart disease (19, 5.5%), and arrythmia (1, 0.3%). The most reported etiology of HF was ischemic (100 trials, 56.5%), followed by genetic (38, 21.5%), other forms of HF (7, 4.0%), and HF with preserved ejection fraction (3, 1.7%), while 29 trials did not specify HF type (16.4%). Other forms of HF included heart failure caused by anthracyclines, Chagas cardiomyopathy, and amyloidosis-related cardiomyopathy. Most trials studied adults (18 years or older), whilst 37 studies included children (younger than 18 years). Of these 37 studies, 24 cell therapy trials, one gene therapy and one tissue engineering trial focused exclusively on children. Regarding the inclusion criteria for participant sex, 336 trials were not limited to a specific sex. Two trials included only male participants, while one trials included only females. In four trials age-range was not provided and in four trials the participant sex was not specified. Most registered trials were of North American (36.4%) or European (31.2%) origin, followed by Asia (24.2%).

**Table 1:** Baseline data and demographics of regenerative cardiac therapy clinical trial registries subdivided for cell therapy, gene therapy, and tissue engineering

		All trials	Cell therapy	Gene therapy	Tissue engineering
Registered trials, n (%)		343	284 (82.8)	40 (11.7)	19 (5.5)
Year of registration, x̄ (Q1-Q3)		2012 (2008–2016)	2011 (2008–2016)	2013 (2007–2019)	2017 (2014–2020)
Condition, n (%)	Coronary artery disease	56 (16.3)	39 (13.7)	16 (40.0)	1 (5.3)
	Myocardial infarction	90 (26.2)	85 (29.9)	2 (5.0)	3 (15.8)
	Heart failure	177 (51.6)	143 (50.4)	21 (52.5)	13 (68.4)
	Congenital heart disease	19 (5.5)	17 (6.0)	0 (0.0)	2 (10.5)
	Arrythmia	1 (0.3)	0 (0.0)	1 (2.5)	0 (0.0)
Phase, n (%)	1	93 (27.1)	71 (25.0)	12 (30.0)	10 (52.6)
	1&2	73 (21.3)	58 (20.4)	11 (27.5)	4 (21.1)
	2	113 (32.9)	101 (35.6)	11 (27.5)	1 (5.3)
	2&3	24 (7.0)	20 (7.0)	3 (7.5)	1 (5.3)
	3	28 (8.2)	25 (8.8)	3 (7.5)	0 (0.0)
	4	2 (0.6)	2 (0.7)	0 (0.0)	0 (0.0)
	N/A	10 (2.9)	7 (2.5)	0 (0.0)	3 (15.8)
Status, n (%)	Completed	158 (46.1)	137 (48.2)	13 (32.5)	8 (42.1)
	Not yet recruiting	6 (1.7)	4 (1.4)	1 (2.5)	1 (5.3)
	Recruiting	36 (10.5)	25 (8.8)	8 (20.0)	3 (15.8)
	Active, not recruiting	14 (4.1)	11 (3.9)	3 (7.5)	0 (0.0)
	Suspended	4 (1.2)	4 (1.4)	0 (0.0)	0 (0.0)
	Terminated	43 (12.5)	39 (13.7)	4 (10.0)	0 (0.0)
	Withdrawn	17 (5.0)	13 (4.6)	4 (10.0)	0 (0.0)
	Unknown	65 (19.0)	51 (18.0)	7 (17.5)	7 (36.8)
Industry-affiliated, n (%)		128 (38.3)	135 (39.4)	97 (34.2)	29 (72.5)
Funding unknown, n (%)		3 (0.9)	3 (0.9)	3 (1.1)	0 (0.0)
Has results, n (%)		111 (32.4)	97 (34.2)	11 (27.5)	3 (15.8)
Estimated number of participants, x̄ (Q1-Q3)		68 (15–90)	69 (20–90)	78 (10–112)	35 (10–48)
Participant age, x̄ (Q1-Q3)	Minimum age	18.5 (18.0–20.0)	18.1 (18.0–20.0)	19.9 (18.0–19.0)	20.7 (18.0–27.5)
	Maximum age	77.7 (75.0–86.0)	78.2 (75.0–85.0)	82.8 (75.0–99.0)	76.4 (75.0–80.0)
Continent of origin,	Asia	83 (24.2)	72 (25.4)	4 (10.0)	7 (36.8)
n (%)	Australia	3 (0.9)	1 (0.4)	1 (2.5)	1 (5.3)
	Europe	107 (31.2)	94 (33.1)	7 (17.5)	6 (31.6)
	North America	125 (36.4)	96 (33.8)	25 (62.5)	4 (21.1)
	South America	18 (5.2)	16 (5.6)	1 (2.5)	1 (5.3)
	Unknown	7 (2.0)	5 (1.8)	2 (5.0)	0 (0.0)

*N/A* not applicable

### Registration Trends Over Time

The earliest registered clinical trial that was retrieved was focused on gene therapy for coronary artery disease and was started in 2001. From 2003 onwards, the number of registered clinical trials increased and reached its peak in 2011, during which most clinical trials were registered (27 trials) (supplementary figure 1). The mean number of yearly registered trials was 15 ± 6 trials. For cell therapy, the mean number of yearly registered trials was 13 ± 6 trials, for gene therapy 2 ± 1, and for tissue engineering 1 ± 1 trial. The first trial using engineered tissue was registered in 2009, the next in 2011, and from 2014 onwards trials investigating engineered tissue were registered yearly.

In 2001 and 2002 coronary artery disease was the most researched condition, surpassed by myocardial infarction in 2003. From 2005 onwards, there is a steep increase in heart failure trials, becoming the most targeted condition from 2009 onwards, surpassing myocardial infarction.

### Trial Status

Of all trials, 158 (46.1%) were registered as completed and 56 (16.3%) were registered as ongoing (recruiting, not yet recruiting or active). A total of 64 (18.6%) trials were registered as terminated (43, 12.5%), withdrawn (17, 5.0%), or suspended (4, 1.2%), while the status of 65 (19.0%) trials was unknown. When comparing cell therapy, gene therapy and tissue engineering, there were no differences in percentage of completed trials (p = 0.163) or in the number of trials terminated, withdrawn, or suspended (p = 0.099) (Table 2).

**Table 2:** Comparison of regenerative medicine categories and funding categories

	Cell therapy	Gene therapy	Tissue engineering	X^2^	*p*-value
Completed	137 (48.2)	13 (32.5)	8 (42.1)	3.62	0.163
Completed amongst non-active trials^a^	137/244 (56.1)	13/28 (46.4)	8/15 (53.3)	0.98	0.613
Terminated/Withdrawn/ Suspended	56 (19.7)	8 (20.0)	0 (0.0)	4.61	0.099
Results available	97 (34.2)	11 (27.5)	3 (15.8)	3.23	0.199
Industry-affiliated	97 (34.5)	29 (72.5)	9 (47.4)	21.59	** < 0.001**^**b**^
	Industry-affiliated	Not industry-affiliated		X^2^	*p*-value
Completed	56 (41.5)	100 (48.8)		1.75	0.186
Terminated/Withdrawn/ Suspended	29 (21.5)	35 (17.1)		1.04	0.309
Results available	42 (31.1)	68 (33.2)		0.16	0.691

Number of trials in tested and column category/Number of trials in column category (%)

^a^Active, recruiting, and trials not yet recruiting were excluded

^b^For industry-affiliated trials, the significant difference between the regenerative medicine categories arises between cell therapy and gene therapy (X^2^ = 21.18, *p* < 0.001). There was no difference between cell therapy and tissue engineering (X^2^ = 1.29, *p* = 0.257) or gene therapy and tissue engineering (X^2^ = 3.55, *p* = 0.060)

For the terminated, withdrawn, and suspended trials, 53/64 (82.8%) provided an explanation for their status (Table 3). The most frequently reported reasons were recruitment difficulties (32.8%) and funding constraints (21.9%). In other trials the decision for terminating, withdrawing, or suspension were based on study data, including method inefficacy or necessary protocol alternation (3 trials), committee recommendation (2 trials), and safety issues (1 trial). Incontinuity of the research team resulted in termination of 3 trials, another 3 trials were never started. For 2 trials, termination was a sponsor decision; both trials were cell therapy trials. Other reasons included unspecified administrative reasons, the COVID-pandemic, political pressure, and laboratory contamination. In 11 (17.2%) trials, no explicit reason for their non-completion was reported. These trials were all cell therapy trials.

**Table 3:** Reasons for terminating, withdrawing, or suspending a clinical trial

Reason for terminating/withdrawing/suspending	All trials	Cell therapy	Gene therapy	Tissue engineering
Recruitment issues, n (%)	21 (32.8)	21 (37.5)	0 (0.0)	0 (0.0)
Funding issues, n (%)	14 (21.9)	12 (21.4)	2 (25.0)	0 (0.0)
Data-based decision^a^, n (%)	6 (9.4)	4 (7.1)	2 (25.0)	0 (0.0)
Incontinuity of research team, n (%)	3 (4.7)	3 (5.4)	0 (0.0)	0 (0.0)
Never started, n (%)	3 (4.7)	1 (1.8)	2 (25.0)	0 (0.0)
Sponsor decision, n (%)	2 (3.1)	2 (3.6)	0 (0.0)	0 (0.0)
Other^b^, n (%)	4 (6.3)	4 (7.1)	0 (0.0)	0 (0.0)
Not specified, n (%)	11 (17.2)	9 (16.1)	2 (25.0)	0 (0.0)

^a^Method inefficacy or protocol alternation, committee recommendation, and safety issues

^b^Administrative reasons, COVID, political pressure, and laboratory contamination

Results were available for 111 trials (32.4%) (Table 1), which included results attached to the trial registration and journal article links with an according NCT-number. When excluding currently ongoing (active, recruiting, not yet recruiting) trials, 181/287 (63.1%) not-ongoing trials had no results available. There was no difference in the availability of results between the three regenerative medicine categories (p = 0.199) (Table 2).

### Study Phases

Amongst all trials, 88.3% were registered as phase one or two, with 10 registrations indicating the trial phase as not applicable without further explanation. For 97 trials, a combined trial phase (phase 1 & 2 or phase 2 & 3) was reported (Table 1). For cell therapy 129/284 (45.4%) trials were in phase one and 121 (42.6%) trials in phase two. For gene therapy this was 23/40 (57.5%) and 14 (35.0%), respectively. In the field of tissue engineering most trials were in phase one (14/19 trials, 73.7%), and only two trials in phase two (2 trials, 10.5%).

A total of 28 trials were registered as phase three and only two trials were in study phase four, both in the field of cell therapy as illustrated in supplementary figure 2.

### Study Funding and Collaboration

In 135 (39.4%) trials, the trial was (partially) funded by an industrial partner. Industry funding and collaboration was more prevalent in the field of gene therapy (29/40 trials, 72.5%) compared to cell therapy (97/284 trials, 34.2%, p < 0.001). There were no significant differences in the number of industry-affiliated trials between gene therapy and tissue engineering (9/19 trials, 47.4%), and between tissue engineering and cell therapy. When comparing industry-affiliated and non-industry-affiliated trials, no differences were observed in the percentage of completed trials (p = 0.186), terminated, withdrawn, or suspended trials (p = 0.309), and trials reporting results (p = 0.691) (Table 2).

### Outcome Measures

Primary outcome measures and means of outcome assessment are summarized in Table 4. Of all trials, only three trials (0.9%) did not report a primary outcome, while 239 (69.7%) reported a single primary outcome and 101 (29.4%) reported multiple primary outcomes. In 135 trials, the primary outcome was a composite endpoint, with different variables as part of the composite. Outcome and outcome assessment was highly variable among identified trials. When categorizing the primary endpoints, 186 trials (54.2%) assessed efficacy and 121 trials assessed safety (35.3%). A total of 35 trials (10.2%) assessed both.

**Table 4:**
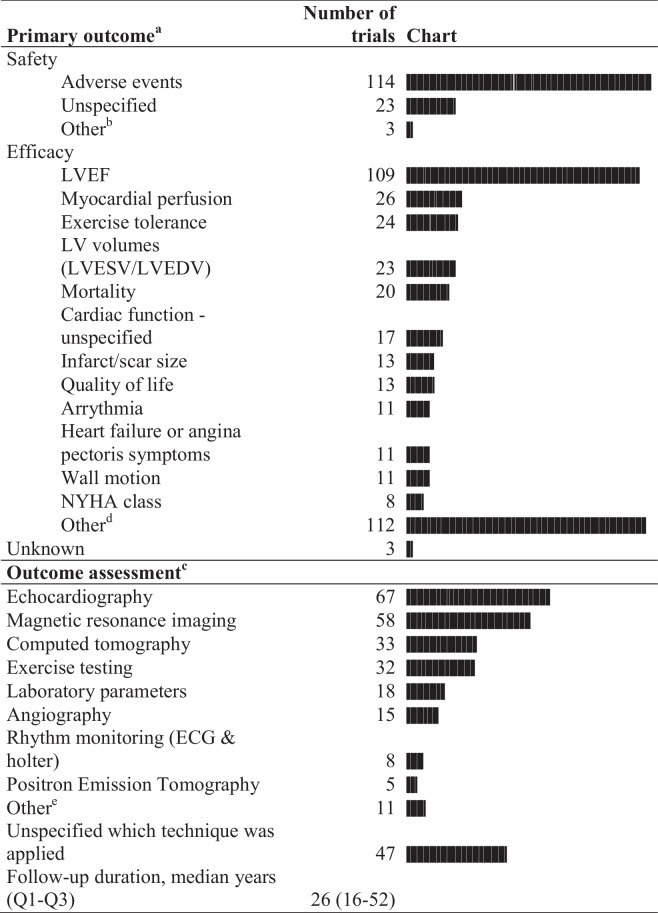
Primary outcome measures and measurement characteristics

Abbreviations: *LVEF* left ventricle ejection fraction (%), *LVESV* left ventricular end systolic volume, *LVEDV* left ventricle end diastolic volume, *NYHA* New York heart association, *ECG* electrocardiogram

^a^101 trials registered multiple outcome measures

^b^Other safety definitions included progression in coronary atherosclerosis, the need for vasoactive medication, and the maximum tolerated dose of intervention

^c^184 trials registered the use of imaging or laboratory testing as measurement modality

^d^Supplementary Table 1A

^e^Supplementary Table 1B

Safety was most often measured through the incidence of adverse events (114 trials). Of these, a total 70 trials (63%) specified which adverse events were assessed, which yielded 50 different adverse event definitions. In 44 trials the adverse events were not specified. In 23 trials it was not specified which outcome parameter was assessed for safety.

The most frequently used primary outcome for determining therapeutic efficacy, was left ventricular ejection fraction (LVEF; 109/186, 58.6%), followed by myocardial perfusion (26 trials, 14.0%), exercise tolerance (24 trials, 12.9%), left ventricular dimensions (23 trials, 6.7%), and mortality (20 trials, 11.0%). A total of 58 other outcome measures were reported, which are listed in supplementary table 1a.

**Fig. 2:**
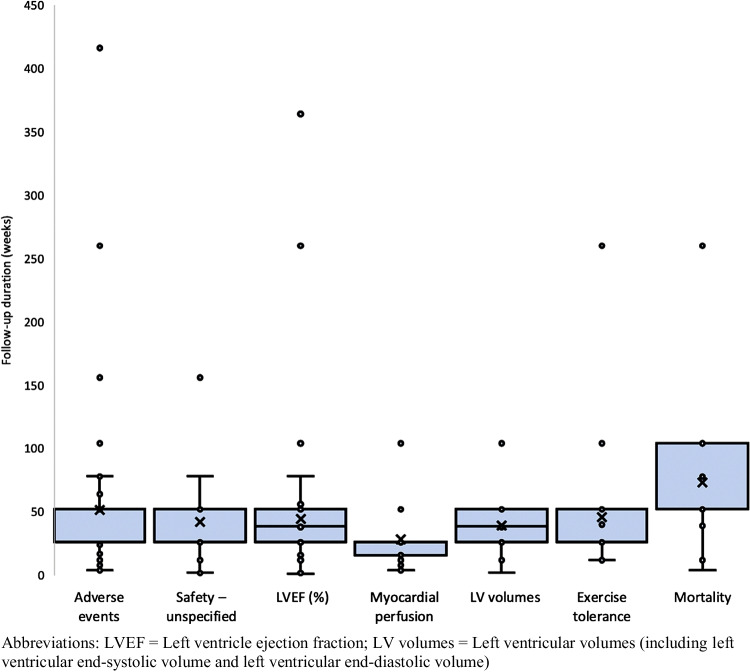
Time upon endpoint measurement in weeks for the 7 most prevalent outcome measures

For outcome assessment, most trials used echocardiography (67 trials), followed by magnetic resonance imaging (58 trials) and computed tomography (33 trials). A total of 32 trials used exercise testing to assess the primary outcome. In 47 trials, it was not specified how outcome assessment was conducted. The median follow-up until endpoint measurement was six months (Q1-Q3: 4 months – 1 year). Nonetheless, there was variability among length of follow-up, ranging from one day up to eight years, as illustrated in Fig. 2. Time until primary outcome assessment was specified in 331 (96.5%) registered clinical trials, the remainder did not specify at which interval the outcome was assessed.

### Cell Therapy

In cell therapy trials, different cell types were investigated, which originated from various regions of the human body (supplementary table 2). Most investigated cell types were non-cardiac specific bone-marrow derived cells (130 trials, 45.8%), while 29 trials (10.2%) investigated cardiac-associated cells (supplementary table 2). The other trials investigated cells which were less differentiated and not specifically derived from heart tissue or prepared to become a cardiomyocyte, such as umbilical cord- (24, 8.5%), adipose tissue- (17, 6.0%), and skeletal muscle-derived (5, 1.8%) cells. Within the category of umbilical cord-derived cells, 8 out of 24 trials specified that the cells were Whartons’ Jelly-derived. In 77 (27.1%) clinical trials, cell type was not specified.

Most of the utilized cells were of autologous origin (67.3%). Whether the cells were harvested from the treated patient or from a donor was not specified in 26 (9.2%) trials (supplementary table 2).

A heterogeneity in cell therapy administration route nomenclature was observed, with 29 different identified terms (supplementary table 3). However, upon categorization, the majority could be grouped into three main routes of administration, being intracoronary, intramyocardial, and systemic injection (supplementary figure 3). Intramyocardial administration routes can be subdivided into catheter-based (endovascular) and syringe-based (by direct exposure of the heart during cardiac surgery).

### Gene Therapy

A total of 40 trials investigated gene therapy for heart disease (supplementary table 4). Vectors for delivery could be divided into viral and non-viral vectors. In total, 27 (67.5%) trials used viral vectors, of which 13 used the adenovirus and 14 used an adeno-associated virus. Non-viral vectors were used in 10 (25.0%) trials, of which 7 used plasmid DNA, 2 hybrid DNA (recombinant DNA molecules), and 1 lipid nanoparticles. The applied vector was not specified in 3 trials.

Genes delivered included genes encoding for vascular endothelial growth factors (13 trials, 32.5%), fibroblast growth factors (4, 10.1 = 0%), hepatocyte growth factors (4, 10.0%), SERCA2a (7, 17.5%), fraxatin (2, 5.0%), adenylyl cyclase type 6 (2, 5.0%), stromal cell-derived factor 1 (2, 5.0%), protein phosphatase inhibitor (2. 5.0%), lysosome-associated membrane protein (1), hERG potassium channel (1), human plakophilin-2a, myosine binding protein C3, and 1 trial describing a CRISPR/Cas9 gene editing system which results in transthyretin reduction.

Administration routes of gene therapy included intramyocardial injection (16 trials, 40%), intracoronary infusion (16 trials, 40%), systemic administration (6 trials, 15%), retrograde infusion in the coronary sinus (1 trial), and one trial with an unknown administration method. Gene therapies aimed at growth factors were mainly administered intramyocardially (71.4%) instead of intracoronary/systemically (28.6%). Trials in which the gene therapy was administered through intracoronary infusion or systemically mainly used a viral vector (21/22 trials, 95.5%), while trials which used intramyocardial injection as administration route less often used viral vectors (5/16, 31.3%).

### Tissue Engineering

Constructed bioartificial tissue with cells or growth factor, in the form of a micrograft, patch or sheet, were investigated in 11 trials (57.9%). The other trials investigated hydrogel-based therapies (5 trials, 26.3%), engineered heart muscle (1 trial, 5.2%), a tissue engineered vascular graft (1 trial, 5.2%), and cells in sprayed form (1 trial, 5.2%) (supplementary table 5).

Regarding administration route, direct placement on the heart was the most prevalent (13 trials, 68.4%), followed by intramyocardial injection (5 trials, 26.3%) and a cell spray (1 trial, 5.2%).

## Discussion

Clinical trials play a pivotal role in evaluating the safety and efficacy of emerging cardiac regenerative interventions aimed at treatment of heart disease. Our study aimed to investigate the focus and fate of all registered clinical trials within regenerative cardiac medicine, with the purpose of exploring the potential role of publication bias (or trial-completion bias), how published and unpublished research affects the field, and to draw lessons and recommendations for future clinical trials. Our main findings include that only a third of all registered trials has yielded results and that a significant number of trials are not completed. Furthermore, we identified major variability in study design, study phase, funding, specific therapies used, primary outcome measures and methods of outcome assessment. Such heterogeneity complicates comparison of effectiveness of different interventions and might hinder the accumulation of evidence needed for meta-analyses and translation of promising experimental therapies to clinical practice.

As mentioned, a significant number of trials are not completed or yield no results. Trials that were not completed were mostly terminated, withdrawn, or suspended, with 17% of trials not providing an explanation to why they remain uncompleted. This is important information, as it might yield relevant lessons for future clinical trials to prevent repeated mistakes in trial design and execution. Other important reasons for discontinuing a trial were recruitment and funding issues. Recruitment issues pose an important challenge, as one looks for a study population that is comparable to the target population, while still homogeneous enough to limit variation. The interventions studied are invasive and possible complications could make potential candidates apprehensive to participate. On the other hand, patients might not be willing to participate in a trial that might sort them in a control arm.

Most trials are registered as either phase one or phase two studies, with only two cell therapy trials advancing to phase four. This might be due to the novelty of these interventions (as seems to be the case for tissue engineering); however, this could also be attributed to decreased interest or lack of progress within the field. On the other hand, it might also simply be due to lack of therapeutic efficacy, or it might be related to funding issues, since regenerative therapies are associated with high costs and protection of intellectual property might be an important obstacle. Whatever the reason, progression into confirmatory phases is essential for regenerative cardiac therapy to move beyond the early stages of investigation and become a clinically applicable treatment option. Improving homogeneity on multiple levels among registered trials could be an important contributory step in achieving this. However, past initiatives to improve uniformity within cardiovascular regenerative medicine have demonstrated that this is easier said, than done [9, 10].

Working from the assumption that uniformity needs to be improved, a major target to improve homogeneity are primary outcomes. Among the included trials this is reflected by the wide array of primary outcomes assessed, methods for outcome assessment and follow-up intervals used in the different trials. While this variation may enable complementary findings across different studies and populations, it limits comparative analysis of results and accumulation of evidence [5].

As an example, over a third of all clinical trials reported the use of composite endpoints with different compositions. Furthermore, a significant portion of trials did not specify the exact composition of their endpoint. For instance, several studies used Major Adverse Cardiovascular Events (MACE) without specifying its exact composition. The lack of consistency is a known problem associated with MACE [11]. Composite endpoints are used to increase statistical power for studies with limited sample sizes and short follow-up periods. Furthermore, they can provide a more clinically relevant assessment of efficacy than single endpoints, especially in heart disease where multiple clinical outcomes may be affected by the same treatment [12, 13]. However, the included endpoints must be of similar importance to patients, have similar frequencies of occurrence, and be affected similarly by the studied treatment [12, 14]. When comparing studies that use composite endpoints with differing compositions or do not specify the composition at all, the purpose of using composite endpoints is defeated and no reliable conclusions can be drawn from study comparison. It is therefore essential to specify and standardize the composition of a used composite endpoint, including follow-up and method of outcome assessment [11]. These standardized endpoints could be adjusted to suit the purpose of each specific clinical phase, determining safety or effectiveness of therapies. To further improve the selected composite endpoints, the recently introduced statistical concept of win ratios for reporting of composite endpoints might be of additional benefit [15]. Furthermore, the number of phases that can be registered for an individual trial may need to be limited to assure that a trial only investigates endpoints relevant to that specific phase, whether it’s safety, or efficacy. At this point, too many individual trials register multiple phases with conflicting endpoints and study designs, which complicates the interpretation of the collected results.

According to a recent position paper, cardiac regenerative therapy serves two complementary strategies, being: (1) inducing exogenous regenerative responses, in which implanted products, cells or tissues are used to replace the structural integrity of damaged myocardial tissue; and (2) stimulation of endogenous regenerative responses, in which the delivered products are aimed at stimulating the endogenous reparative processes [9]. Generally speaking, cell therapy and tissue engineering can encompass both strategies, while gene therapy is mainly used to stimulate endogenous responses aimed at restoration of the damaged tissue.

Numerous studies have been conducted on cellular therapies and have collected a wealth of data on various types of cells sourced from different origins. Most cell therapy trials have been conducted using so-called “first generation” cells, derived from bone marrow and peripheral blood, or other mesenchymal tissues (adipose tissue, umbilical cord, etc.). Due to their mesenchymal origin, these usually consist of a heterogeneous cell population with a relatively limited regenerative potential, suggesting that these cells have a modest direct effect on cardiac function. However, they can serve as useful tools to stimulate endogenous regenerative responses through paracrine influences. On the other hand, the “second generation” cell lines consist of more cardiac-specific purified cell populations with a presumable greater regenerative potential that can be used for an exogenous regenerative approach. While first generation cell lines are usually hampered by their limited differentiation potential, second generation cell lines, while more cardiac-specific, are associated with low yield and extensive culture periods to obtain sufficient numbers for transplantation. A more detailed review on their distinctive characteristics, has been published elsewhere [16, 17]. Whatever their origin, cell-based therapies have generally been hampered by problems related to engraftment and retention, poor survival and failure to differentiate, which limits their therapeutic efficacy [9, 17, 18]. Due to the inherent heterogeneity of cell populations combined with the aforementioned challenges related to engraftment, it is of utmost importance for cell therapy research to adopt consistent and standardized protocols and terminology in order to facilitate clear scientific communication and efficiently progress the field. However, as our data demonstrates, this currently seems to be lacking, especially relating to administration routes and strategies, which are of particular importance for cell therapy given their engraftment-related difficulties, making it difficult to draw reliable conclusions [9, 18].

Tissue engineering could theoretically provide an outcome for a main obstacle within cell therapy: being the low retention rate of cells in target tissues [19]. Cardiac tissue engineering aims to remuscularize myocardial scars, provide paracrine support and form a substitute tissue for failing hearts [9]. For better cell retention, different complex approaches are being assessed, including the use of hydrogels, sheets, extracellular matrix, and bioartificial tissues. Tissue engineering involves not only the selection of appropriate cells, but also the composition of a tissue-like structure in which these transplanted cells can survive and develop into functional heart tissue. Several studies are ongoing due to the more recent popularity of tissue engineering. Especially for such complex techniques, comparison between studies could strengthen the scientific evidence for tissue engineering techniques. In these cases, uniform trial design, standardized outcomes and patient selection are important issues to assess. Although promising, issues regarding cell survival and engraftment still play a role, as well as safety concerns regarding rejection, arrhythmia and tumour development, since these cells are embedded in a protective matrix that could enhance the risk of side effects [9].

For cardiac gene therapy, delivery is key to a successful treatment [20]. The efficacy of treatment is dependent on the vector used for delivery. Currently, potential vectors can be subdivided into viral and non-viral vectors, all with their own specific strengths and weaknesses. Non-viral vectors include liposomes, while viral vectors include adenoviruses and adeno-associated viruses [8, 20, 21]. Current challenges to overcome are mainly related to delivery, cardiac specificity of vectors used and immune system evasion or modulation to improve efficacy of treatment [8, 20, 21]. As indicated by our results, a multitude of genes have been investigated in the registered clinical trials, with a multitude of vectors and delivery routes. Furthermore, gene therapy is currently mostly studied for coronary artery disease and heart failure, which is also reflected by the specific genes delivered (i.e., to stimulate angiogenesis, remodeling, or replacement of a defective gene). Besides gene delivery, gene-editing approaches using CRISPR/Cas9 might also be of interest since this would facilitate repair of a defective gene instead of replacement [4, 8, 19, 22].

With inconsistent study results and a wide variety of trials not progressing to later phases, the field of regenerative cardiac therapy appears stagnating, with issues addressed above as important contributory factors [8]. To address these, there is a growing need for establishment of standardization across all stages of clinical trial development and execution. Furthermore, one might wonder whether there should be a more active role regarding (proactive) surveillance of trial status by clinical trial registries. Subsequently, the question arises who should be responsible for the oversight and standardization guidelines, and if this should be based on guidelines by professional cardiovascular societies such as the European Society of Cardiology, or the American Heart Association. As a starting point and based on our observations, we formulated the following recommendations to improve the conductance and registration of clinical trials within the field of regenerative cardiac therapy. These recommendations transcend individual indications (such as myocardial infarction vs. heart failure) and should be applicable to all categories of regenerative cardiac medicine.

1. Accurate and transparent registration and regular updating of study status and design is essential for an accurate overview of the current field. Furthermore, specification of mutations in study status (i.e. providing reasons for discontinuation, suspension) is adamant to prevent repeated mistakes in trial design
2. Registration of a trial should include specification of the study population, including specific disease and stage of disease, in order to reliably assess the effects and safety of a treatment and improve external validity to relevant patient populations
3. Registration of a trial should include specification of the studied intervention, including delivery routes, cell types and origin, timing of intervention after an index event, and duration of therapy. Unambiguous use of terminology with clear descriptions should be applied to avoid confusion
4. Registration of trials should include specification of primary endpoints and duration of follow-up. Ideally, these endpoints are standardized within a specific research field to accommodate the needs for each trial phase, to improve comparability and create a larger collection of usable data, considering the FAIR principles at each phase of the research process
5. Attention to cost-efficiency and valorization potential of cardiac regenerative therapies is necessary to obtain and maintain interest of both private and public partners to ensure consistent and reliable funding of research, from early stages to clinical practice. Furthermore, protection of intellectual property should be ensured

Additionally, technical aspects of the studied therapies currently seem understudied, including challenges regarding delivery or access to the heart. New and innovative ideas might offer a solution and provide a new direction for regenerative cardiac therapy or provide opportunities to overcome the encountered obstacles. One such example might be isolated organ perfusion, which can be conducted both in vivo and ex situ and has allowed for the study of regenerative therapies in lungs, kidneys, and livers [23–25], circumventing the challenges associated with systemic delivery [20, 23]. When focusing on heart disease, most clinical trials are hampered by concerns surrounding delivery, preliminary clearance by other organs, toxicity, inflammation, and oncogenesis when administered systemically. These issues can be overcome when the therapeutic interventions (i.e., cell therapy, gene therapy) are applied during isolated organ perfusion. By isolating the organ in a metabolically and immunologically favorable condition, it is possible to directly investigate and manipulate the factors that influence important obstacles related to uptake and delivery that were encountered during in vivo clinical trials and potentially improve the success rate of regenerative cardiac therapy.

The main limitation to this paper is primarily related to the quality of clinical trial registration, as indicated by the relatively high number of studies that have an unknown study status, which obscures our ability to provide an accurate overview of the field [4, 8].

Furthermore, we did not conduct an analysis of all the published results and did not draw a comparison between published and unpublished research. However, we consider this beyond the scope of this paper, since a multitude of papers have been written on the summarized results of these published clinical trials. We therefore opted to focus on the earlier phase of clinical trial registration to provide a full overview of the current status of regenerative cardiac therapy.

In conclusion**,** a large number of registered clinical trials are currently not completed or yield no results. Furthermore, progress in regenerative cardiac therapy is currently hampered by heterogeneity in clinical trial design, which complicates comparison and limits the ability to draw meaningful conclusions regarding the (non-)efficacy of certain therapeutic interventions. To enable comparison and evidence-based conclusions of the safety and efficacy of regenerative cardiac therapy, standardization of trials is necessary. Such standardization can help to ensure that study results are reliable and can be generalized to larger populations, ultimately leading to more effective therapies, improved patient outcomes and translation into clinical practice.

## Supplementary Information

Below is the link to the electronic supplementary material.

Supplementary file1 (DOCX 817 KB)

## Abbreviations

HF: Heart failure
LVEF: Left ventricular ejection fraction
MACE: Major adverse cardiovascular events
