# Supplementary material for: Past Trends and Future Directions of Cardiac Regenerative Medicine – A Systematic Analysis of Clinical Trial Registries
Source: J Cardiovasc Transl Res. 2024 Oct 3;18(1):209–20. doi: 10.1007/s12265-024-10563-1 (PMC11885401; doi:10.1007/s12265-024-10563-1)
Supplement: Supplementary file 1 — Supplementary file1 (DOCX 817 KB) [file 12265_2024_10563_MOESM1_ESM.docx]

**Supplementary materials**

Journal of Cardiovascular Translational Research

Past Trends and Future Directions of Cardiac Regenerative Medicine – A Systematic Analysis of Clinical Trial Registries

Maaike Wulfse*^1^ BSc, Mats T. Vervoorn*^1^ MD, Jantijn J.G.J. Amelink^1^ BSc, Elisa M. Ballan^1,2,3^ MSc, Saskia C.A. De Jager^3^ PhD, Joost P.G. Sluijter^3,4^ PhD, Pieter A. Doevendans^2,5^ MD PhD, Peter-Paul M. Zwetsloot^3,5^ MD PhD, Niels P. Van der Kaaij^1^ MD PhD

^*^Both authors contributed equally

^1^University Medical Center Utrecht. Department of Cardiothoracic Surgery. Division of Heart & Lungs. Utrecht, The Netherlands

^2^Netherlands Heart Institute. Utrecht, The Netherlands

^3^University Medical Center Utrecht. Department of Cardiology, Laboratory of Experimental Cardiology. Division of Heart & Lungs. Utrecht, The Netherlands

^4^Regenerative Medicine Utrecht, Circulatory Health Research Center, University Utrecht, Utrecht, The Netherlands

^5^University Medical Center Utrecht. Department of Cardiology. Division of Heart & Lungs. Utrecht, The Netherlands

**Supplementary files**

**Supplementary table 1**

Supplementary table 1A. Other primary outcomes

| **Other primary outcome measures** | **Number** |
| --- | --- |
| Feasibility | 11 |
| Hospitalization (unspecified) | 8 |
| VO2 max | 6 |
| Wall thickness | 5 |
| Heart failure hospitalization | 4 |
| Heart failure (unspecified) | 3 |
| Laboratory parameters (unspecified) | 3 |
| Myocardial viability | 3 |
| VO2 | 3 |
| Clinical events (unspecified) | 2 |
| Coronary atherosclerotic burden | 2 |
| Hypersensitivity | 2 |
| Inflammation | 2 |
| Maximum tolerated dose | 2 |
| Morbidity | 2 |
| Myocardial infarction | 2 |
| Myocarditis | 2 |
| Myocardium metabolic | 2 |
| Number of tolerated LVAD weans | 2 |
| Recurrence of myocardial infarction | 2 |
| RVEDV & RVESV | 2 |
| Tumors | 2 |
| Toxicity | 2 |
| Anti-adenovirus antibodies | 1 |
| Atherosclerosis | 1 |
| Biodistribution of the vector | 1 |
| Cardiac arrest | 1 |
| Clinical stabilization of cardiomyopathy | 1 |
| Complications with drug administration method | 1 |
| ECG parameters | 1 |
| Evaluation of cardiomyocyte histologic correction | 1 |
| Immune sensitization | 1 |
| Intervention for graft failure | 1 |
| Interventions (unspecified) | 1 |
| Intraoperative hemostasis | 1 |
| Ischemic score | 1 |
| LV dimensions | 1 |
| LV filling pressure | 1 |
| MLHFQ score | 1 |
| MSC identification | 1 |
| Myocardial innervation | 1 |
| Myocardial rupture | 1 |
| Pharmacodynamics & pharmacokinetics | 1 |
| Post-operative complication | 1 |
| Procedure success | 1 |
| Pulmonary capillary wedge pressure | 1 |
| Restenosis | 1 |
| Risk of surgery | 1 |
| RVEF | 1 |
| Survival benefit | 1 |
| Temperature | 1 |
| Time to 1 mm ST depression during exercise-stress testing | 1 |
| Time to onset of ischemic ECG changes during ETT | 1 |
| Tolerability (unspecified) | 1 |
| Total atheroma volume | 1 |
| Tricuspid regurgitation | 1 |
| Troponin | 1 |
| Tumor markers | 1 |
| Uncontrolled systemic infection | 1 |
| Unplanned myocardial revascularization surgery/angioplasty | 1 |
| Unstable angina hospitalization | 1 |
| VEGF-levels | 1 |

Supplementary table 1B. Other outcome additional imaging or tests

| **Other outcome additional imaging or tests** |  |
| --- | --- |
| Advanced imaging | 1 |
| Biopsy | 2 |
| Cardiopulmonary testing | 1 |
| Image analyses | 1 |
| Intravascular ultrasound | 2 |
| Scintigraphy | 1 |
| Tc-sestamibi scintigraphy | 1 |
| Thallium scintigraphy | 1 |
| Ventriculography | 1 |

**Supplementary table 2**

Table 2A. Cell types and donor-origin in regenerative cardiac cell therapy

| **Cell type** | **Number (%)** | **Chart** |
| --- | --- | --- |
| Bone marrow-derived | 130 (45.8) | \|\|\|\|\|\|\|\|\|\|\|\|\|\|\|\|\|\|\|\|\|\|\|\|\|\|\|\|\|\|\|\|\|\|\|\|\|\|\|\|\|\|\|\|\|\|\|\|\|\|\|\|\|\|\|\|\|\|\|\|\|\|\|\|\|\|\|\|\|\|\|\|\|\|\|\|\|\|\|\|\|\|\|\|\|\|\|\|\|\|\|\|\|\|\|\|\|\|\|\|\|\|\|\|\|\|\|\|\|\|\|\|\|\|\|\|\|\|\|\|\|\|\|\|\|\|\|\|\|\| |
| Cardiac-associated^a^ | 29 (10.2) | \|\|\|\|\|\|\|\|\|\|\|\|\|\|\|\|\|\|\|\|\|\|\|\|\|\|\|\|\| |
| Umbilical cord-derived | 24 (8.5) | \|\|\|\|\|\|\|\|\|\|\|\|\|\|\|\|\|\|\|\|\|\|\|\| |
| Adipose tissue-derived | 17 (6.0) | \|\|\|\|\|\|\|\|\|\|\|\|\|\|\|\|\| |
| Skelet muscle-derived | 5 (1.8) | \|\|\|\|\| |
| Unspecified | 77 (27.1) | \|\|\|\|\|\|\|\|\|\|\|\|\|\|\|\|\|\|\|\|\|\|\|\|\|\|\|\|\|\|\|\|\|\|\|\|\|\|\|\|\|\|\|\|\|\|\|\|\|\|\|\|\|\|\|\|\|\|\|\|\|\|\|\|\|\|\|\|\|\|\|\|\|\|\|\|\| |
| Other^b^ | 2 (0.7) | \|\| |
| **Donor-origin** | **Number (%)** | **Chart** |
| Autologous | 191 (67.3) | \|\|\|\|\|\|\|\|\|\|\|\|\|\|\|\|\|\|\|\|\|\|\|\|\|\|\|\|\|\|\|\|\|\|\|\|\|\|\|\|\|\|\|\|\|\|\|\|\|\|\|\|\|\|\|\|\|\|\|\|\|\|\|\|\|\|\|\|\|\|\|\|\|\|\|\|\|\|\|\|\|\|\|\|\|\|\|\|\|\|\|\|\|\|\|\|\|\|\|\|\|\|\|\|\|\|\|\|\|\|\|\|\|\|\|\|\|\|\|\|\|\|\|\|\|\|\|\|\|\|\|\|\|\|\|\|\|\|\|\|\|\|\|\|\|\|\|\|\|\|\|\|\|\|\|\|\|\|\|\|\|\|\|\|\|\|\|\|\|\|\|\|\|\|\|\|\|\|\|\|\|\|\|\|\|\|\|\|\|\|\| |
| Allogeneic | 63 (22.2) | \|\|\|\|\|\|\|\|\|\|\|\|\|\|\|\|\|\|\|\|\|\|\|\|\|\|\|\|\|\|\|\|\|\|\|\|\|\|\|\|\|\|\|\|\|\|\|\|\|\|\|\|\|\|\|\|\|\|\|\|\|\|\| |
| Ex vivo cultured | 4 (1.4) | \|\|\|\| |
| Unspecified | 26 (9.2) | \|\|\|\|\|\|\|\|\|\|\|\|\|\|\|\|\|\|\|\|\|\|\|\|\|\| |

^a^A cardiac associated cell was either derived from cardiac tissue or altered to become a cardiomyocyte of cardiomyocyte-progenitor.
^b^Other modalities of cell therapy included extracellular vesicle-enriched secretome of cardiovascular progenitor cells differentiated from induced pluripotent stem cells and autologous mesenchymal stem cell derived exosomes and mitochondria.

Supplementary table 2B. Stem cells utilized in regenerative cardiology registrated clinical trials

| **Cell** | **Number** | **Chart** | |
| --- | --- | --- | --- |
| Unspecified | 91 | \|\|\|\|\|\|\|\|\|\|\|\|\|\|\|\|\|\|\|\|\|\|\|\|\|\|\|\|\|\|\|\|\|\|\|\|\|\|\|\|\|\|\|\|\|\|\|\|\|\|\|\|\|\|\|\|\|\|\|\|\|\|\|\|\|\|\|\|\|\|\|\|\|\|\|\|\|\|\|\|\|\|\|\|\|\|\|\|\|\|\| | |
| AC 133+ | 2 | \|\| | |
| Cardiac-associated | 29 | \|\|\|\|\|\|\|\|\|\|\|\|\|\|\|\|\|\|\|\|\|\|\|\|\|\|\|\|\| | |
| CD 133+ | 15 | | \|\|\|\|\|\|\|\|\|\|\|\|\|\|\| |
| CD 34+ | 18 | \|\|\|\|\|\|\|\|\|\|\|\|\|\|\|\|\|\| | |
| Mononuclear | 45 | \|\|\|\|\|\|\|\|\|\|\|\|\|\|\|\|\|\|\|\|\|\|\|\|\|\|\|\|\|\|\|\|\|\|\|\|\|\|\|\|\|\|\|\|\| | |
| DC | 1 | \| | |
| G-CSF | 5 | \|\|\|\|\| | |
| Ixmyelocel-T | 3 | \|\|\| | |
| Mesenchymal | 70 | \|\|\|\|\|\|\|\|\|\|\|\|\|\|\|\|\|\|\|\|\|\|\|\|\|\|\|\|\|\|\|\|\|\|\|\|\|\|\|\|\|\|\|\|\|\|\|\|\|\|\|\|\|\|\|\|\|\|\|\|\|\|\|\|\|\|\|\|\|\| | |
| Skeletal muscle cell | 5 | \|\|\|\|\| | |
| Mesenchymal stem cell derived exosomes and mitochondria | 1 | \| | |

Supplementary table 2C. Cardiac-associated stem cells utilized in regenerative cardiology registrated clinical trials

| **Cardiac-associated stem cells** |  |
| --- | --- |
| Allogeneic cardiac stem cells |  |
| Allogeneic cardiosphere-derived cells | 4x |
| Allogeneic iPS Cell-derived cardiomyocytes | 2x |
| Allogenic human pluripotent stem cell-derived cardiomyocytes |  |
| Autologous CardAP-cells |  |
| Autologous cardiac progenitor cell transplantation |  |
| Autologous cardiac stem cells |  |
| Autologous cardiac stem cells (JRM-001) | 2x |
| Autologous cardiac stem cells derived from right atrium | 2x |
| Autologous cardiosphere-derived stem cells |  |
| Autologous human cardiac-derived stem cells |  |
| Bone marrow mesenchymal cardiopoietic cells | 3x |
| Bone marrow-derived cardiopoietic cells |  |
| C-kit+ cardiac stem cells |  |
| Cardiac progenitor cells |  |
| Cardiosphere-derived cells |  |
| Extracellular vesicle-enriched secretome of cardiovascular progenitor cells |  |
| Human embryonic stem cells derived cardiomyocytes |  |
| Human Pluripotent stem cell-derived cardiomyocytes |  |
| iPS differentiated cardiomyocytes | 5x |

**Supplementary table 3**

Supplementary table 3. Heterogeneity of terms for cell therapy administration method used in clinical trial registration

| **Cell therapy method specified** |  |
| --- | --- |
| Cell mobilization | 4 |
| Cell transfusion | 1 |
| Endocardial implantation | 2 |
| Endocardial injection | 3 |
| Endomyocardial injection | 5 |
| Epicardial injection | 3 |
| Gelatin Hydrogel Sheet | 1 |
| Infusion into coronary sinus | 3 |
| Injection in left axillary lymph node | 1 |
| Intracardiac cell infusion | 2 |
| Intracardiac administration | 1 |
| Intracoronary administration | 10 |
| Intracoronary infusion | 59 |
| Intracoronary injection | 28 |
| Intracoronary transfer | 5 |
| Intracoronary transplantation | 9 |
| Intramuscular injection | 1 |
| Intramyocardial administration | 4 |
| Intramyocardial implantation | 9 |
| Intramyocardial infusion | 2 |
| Intramyocardial injection | 81 |
| Intramyocardial transplantation | 4 |
| Intrapericardial injection | 1 |
| Intravenous | 21 |
| Intraventricular injection | 1 |
| Laser channels | 1 |
| MyoStar® (Biosense Webster) injection catheter | 1 |
| Single injection of 0.1-0.2 ml around each laser channel | 1 |
| Transendocardial injection | 13 |

**Supplementary table 4**

Supplementary table 4. Gene therapies in clinical trial registries

| **Type** | **Vector** | **Gene/coding for** | **Number of trials** | |
| --- | --- | --- | --- | --- |
| AAV1/SERCA2a | Adeno-associated virus serotype 1 capsid | SERCA2A | | 7 |
| Ad5FGF-4 | Adenovirus 5 | FGF 4 | | 4 |
| AAVrh.10hFXN | Rh.10 adeno-associated virus | Fraxatin | | 2 |
| Ad5.hAC6 | Adenovirus 5 | Adenylyl cyclase type 6 | | 2 |
| VM202RY | Hybrid dna | HGF | | 2 |
| AAV2i8 | Adeno-associated virus serotype 2 synthetic capsid variant | Protein phosphatase inhibitor 1 | | 2 |
| AAV9.LAMP2B | Adeno-associated virus serotype 9 | Lysosome-associated membrane protein 2 isoform B transgene | | 1 |
| AAVRH.74 | Rh. 74 adeno-associated virus | Human Plakophillin-2a | | 1 |
| ACRX-100 | Plasmid DNA | Stromal Cell-Derived Factor 1 (SDF-1) | | 1 |
| Ad-HGF | Adenovirus | HGF | | 1 |
| ADGVVEGF121.10NH | Adenovirus | VEGF 121 | | 1 |
| AdKCNH2-G628S | Adenovirus | Genetic mutation in the KCNH2 gene (hERG potassium channel) | | 1 |
| AdVEGF-All6A+ | Adenovirus | VEGF | | 1 |
| AdVEGF-D | Adenovirus | VEGF D (Dimutase homolog) | | 1 |
| AdVEGFXC1 | Adenovirus | VEGF | | 1 |
| hVEGF165 | Not specified | VEGF 165 | | 1 |
| INXN-4001 | Plasmid DNA | Stromal Cell-Derived Factor 1α, VEGF 165, and S100A1 calcium-binding protein | | 1 |
| NTLA-2001 | Lipid nanoparticles | CRISPR/Cas9 gene editing system | | 1 |
| pVGI.1(VEGF2) | Plasmid DNA | VEGF 2 | | 1 |
| TN-201 | Adeno-associated virus serotype 9 | Myosin binding protein C3 | | 1 |
| VEGF | Not specified | VEGF | | 1 |
| VEGF | Plasmid DNA | VEGF | | 1 |
| VEGF-A165/bFGF | Plasmid DNA | VEGF and FGF | | 1 |
| VEGF-D | Adenovirus | VEGF D (Dimutase homolog) | | 1 |
| VEGF1 | Plasmid DNA | VEGF 1 | | 1 |
| VEGF165 | Not specified | VEGF 165 | | 1 |
| VM202 | Plasmid DNA | HGF | | 1 |

Abbreviations: FGF = Fibroblast growth factor; HGF = Hepatocyt growth factor; VEGF = Vascular endothelial growth factor

**Supplementary table 5**

Supplementary table 5. Tissue engineering in clinical trial registries

| **Investigated tissue engineering** |
| --- |
| ADR-002K administered through cell spray |
| Alginate hydrogel (2x) |
| Algisyl device |
| Allogeneic umbilical cord Wharton’s jelly-derived adult mesenchymal stem cells colonized on human pericardial matrix |
| Atrial Appendage Micrograft Transplant patch |
| Autologous atrial appendage micrografts with fibrin gel |
| Autologous myoblast sheet |
| Collagen patches with adipose-derived stem cells |
| CorMatrix extracellular matrix biomaterial |
| Engineered heart muscle |
| Epicardial delivery of a fibrin patch embedding human embryonic stem cell-derived CD15+ Isl-1+ progenitors |
| Extracellular matrix patch with cultured Wharton’s jelly mesenchymal stem cells |
| Gelatin hydrogel sheet incorporating with basic fibroblast growth factor |
| Heart patch developed from amnion bilayer seeded with amnion epithelial stem cells and patient’s autologous cardiomyocytes |
| Human (allogeneic) iPS cell-derived cardiomyocyte sheet |
| Human umbilical cord-derived mesenchymal stem cells with injectable collagen scaffold |
| Tissue engineered vascular grafts |
| VentriGel |

**Supplementary figure 1**


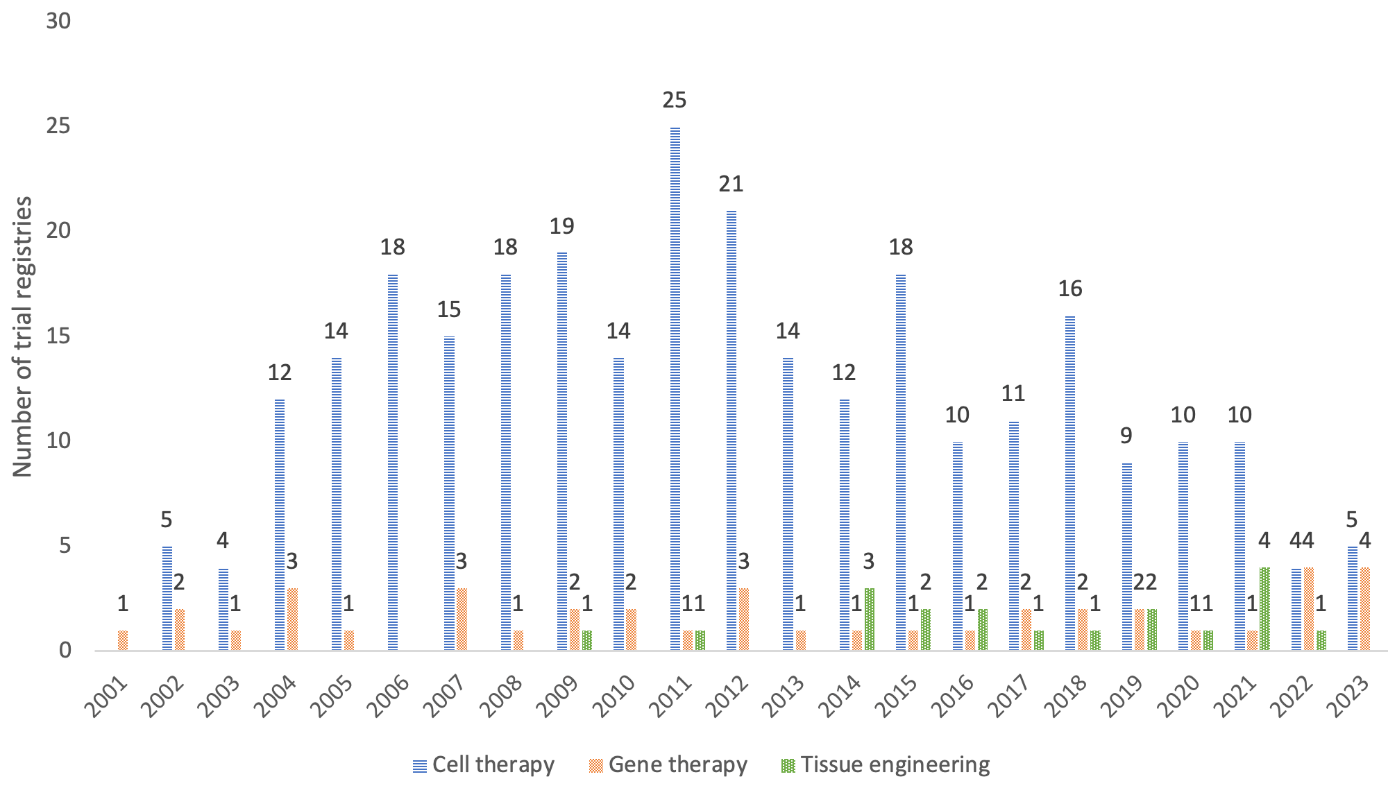


1b

1a


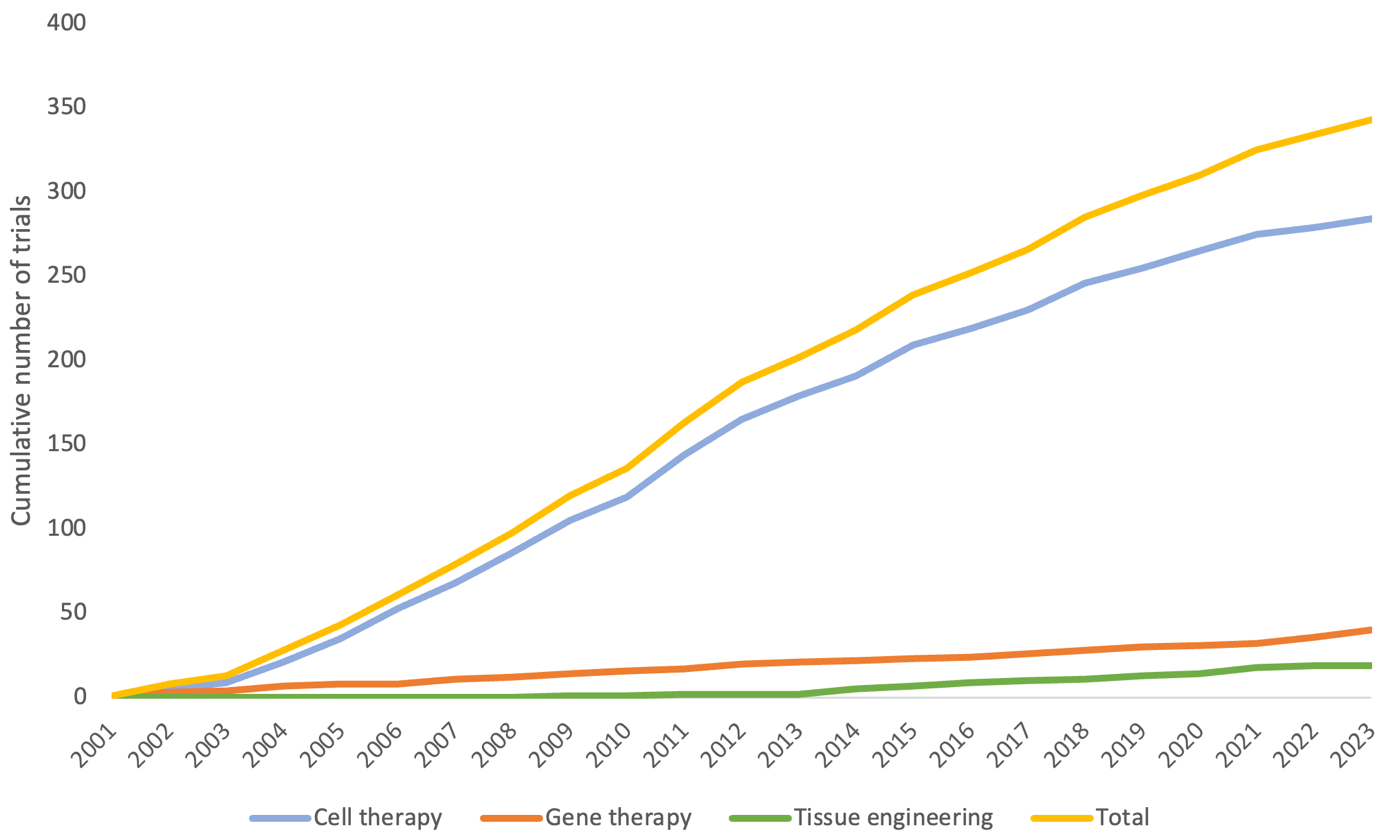


**Supplementary fig. 1** Regenerative cardiology categories over time; 1A: yearly number of trials within the three different regenerative cardiology categories; 1B cumulative number of trials within regenerative cardiology

**Supplementary figure 2**


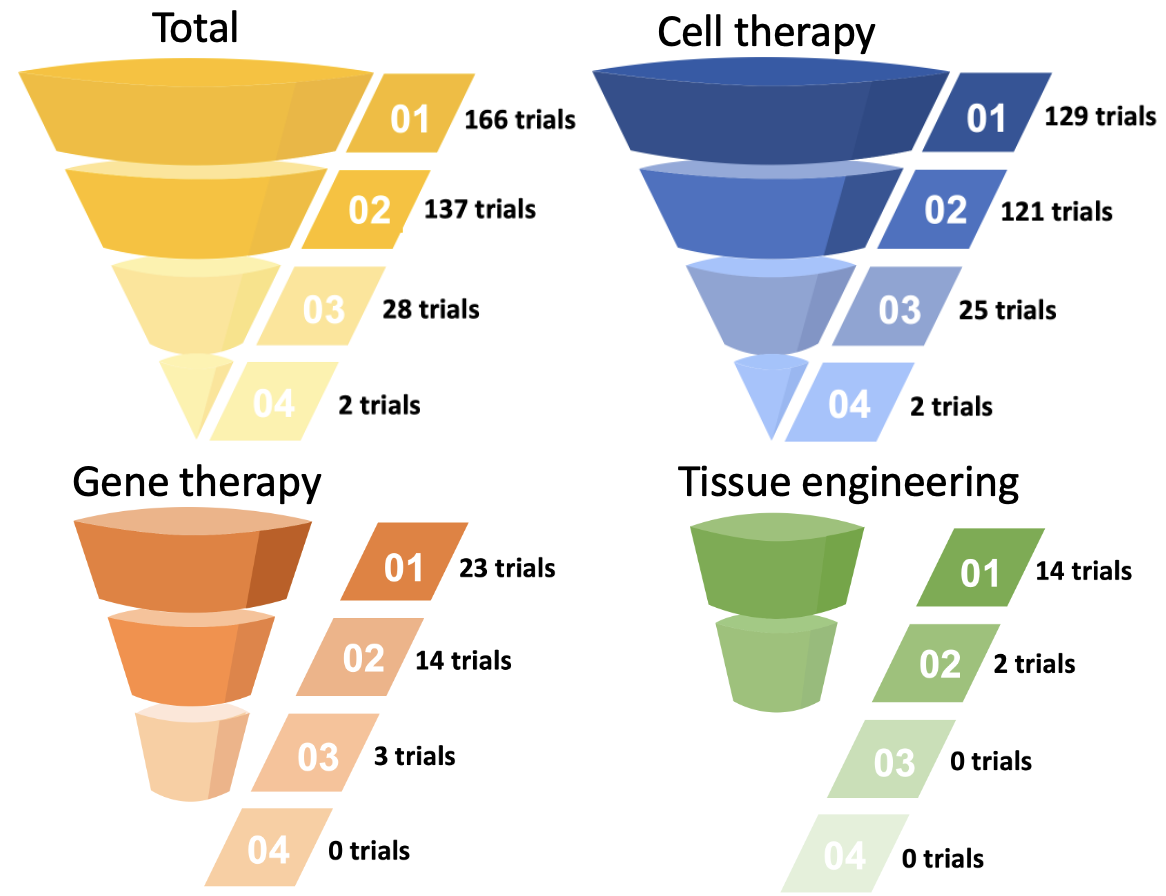


**Supplementary fig. 2** Study phases for all clinical trials and for the individual categories of cell therapy, gene therapy, and tissue engineering

**Supplementary figure 3**


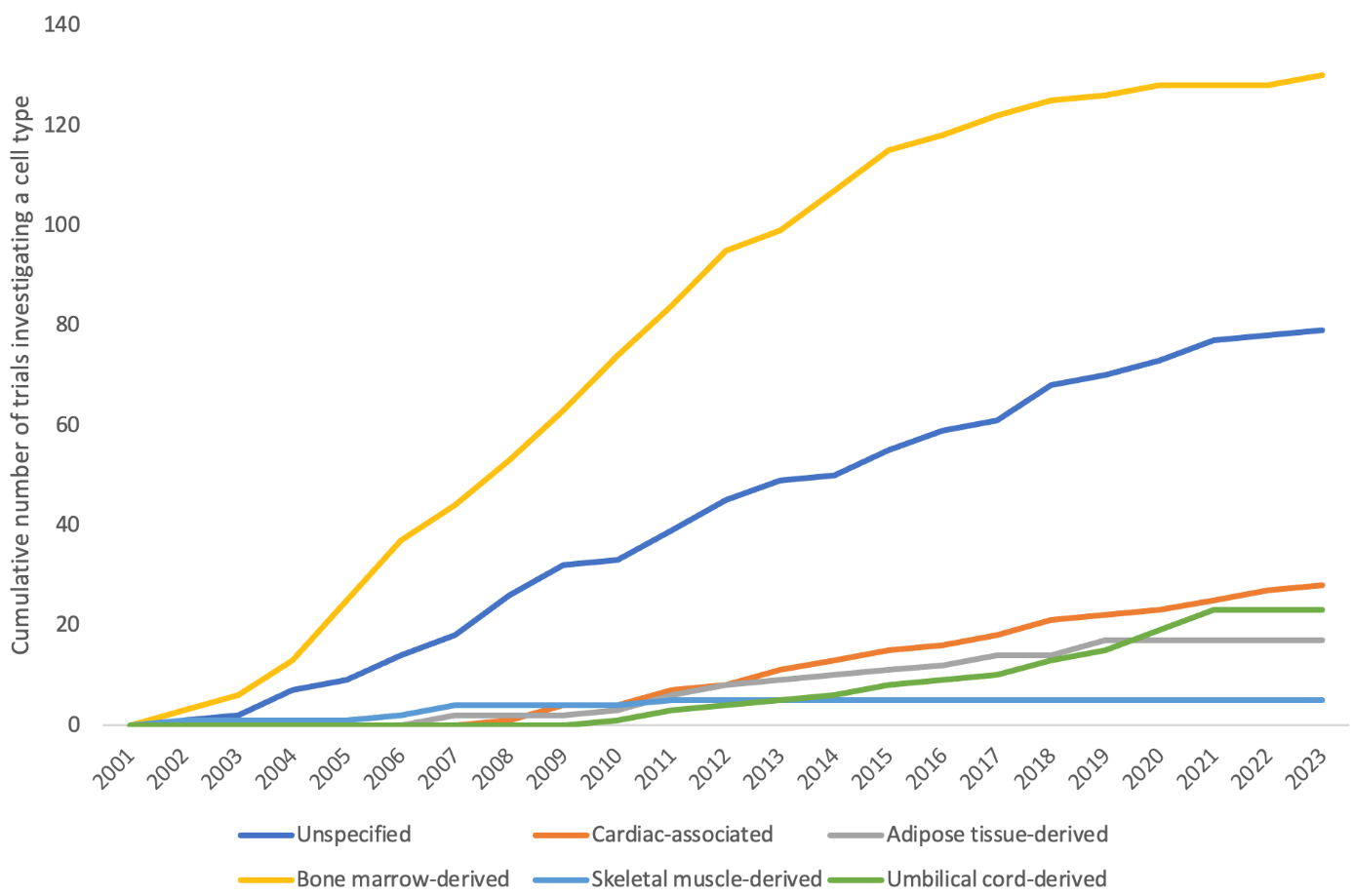


**Supplementary fig. 3** Stem cells used within regenerative cardiac therapy over time

**Supplementary figure 4


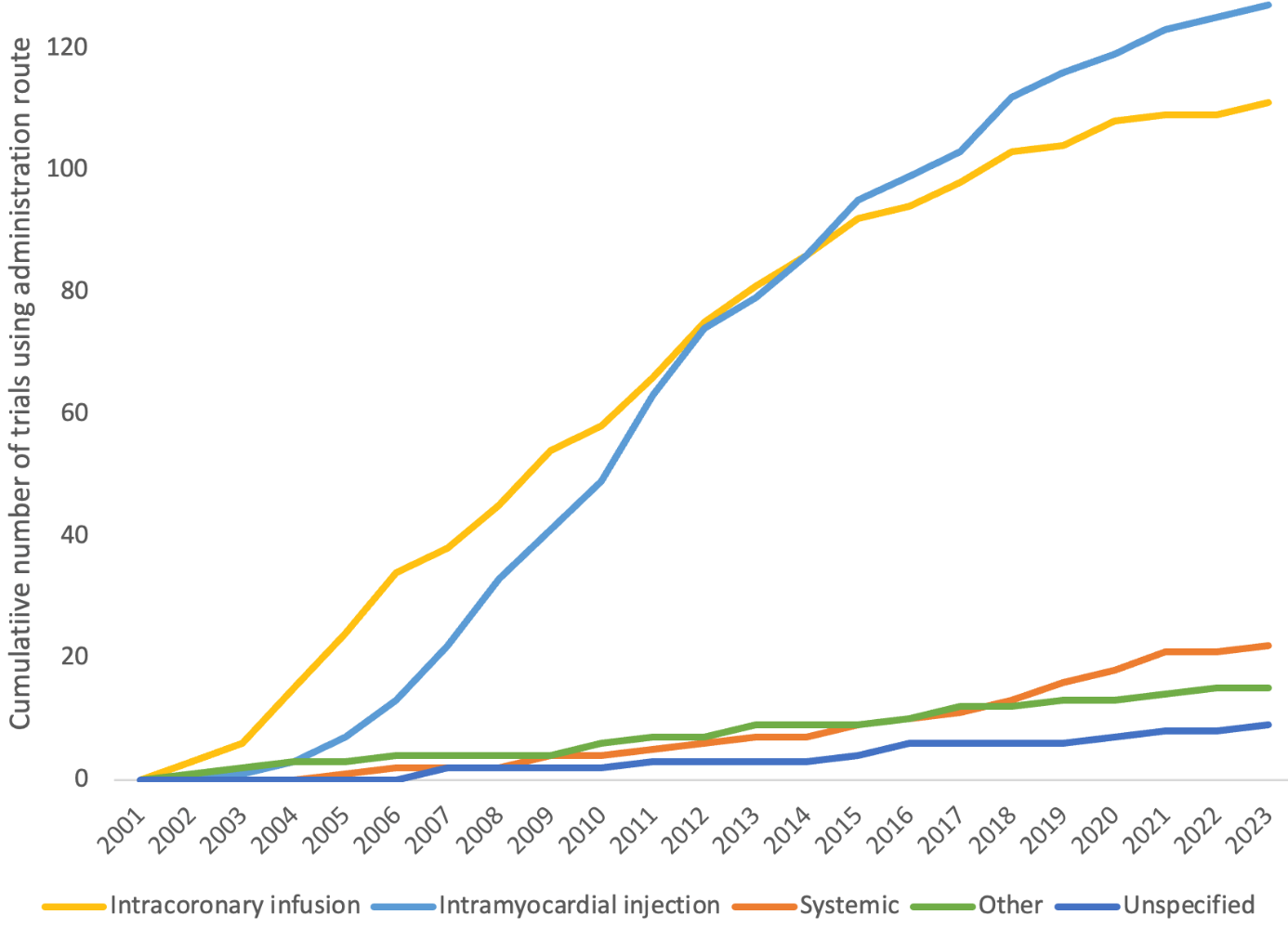
**

**Supplementary fig. 4** Cell therapy administration routes over time.

Other methods of administration included cell mobilization (4 trials), coronary sinus infusion (3 trials), intracardiac infusion (3 trials), laser channel (2 trials), intramuscular injection (1 trial), intrapericardial injection (1 trial), and injection in a left axillary lymph node (1 trial).

**Appendix 1**

*Clinicaltrials.gov*

Regenerative medicine OR cell therapy OR genetic therapy OR gene therapy OR tissue engineering | Heart Diseases OR Heart Failure

*Cochrane libraby/CENTER*

((MeSH descriptor: [Heart Diseases]) OR (MeSH descriptor: [Heart Failure])) AND ((regenerative medicine):ti,ab,kw OR (cell therapy):ti,ab,kw OR (gene therapy):ti,ab,kw OR (tissue engineering):ti,ab,kw)

*EU clinical trials register*

(“heart failure” OR “heart disease”) AND (“gene therapy” OR gene OR cell OR “tissue engineering” OR “genetic therapy” OR “cell therapy” OR “stem cell”)

*World Health Organization International Clinical Trials Registry platform (WHO ICTRP)*

(Heart disease OR heart failure) AND (regenerative medicine OR cell therapy OR gene therapy OR tissue engineering OR genetic therapy)

*Clinicaltrials.gov Tissue Engineering*

Tissue engineering OR biofibrication OR sheet OR hydrogel OR extracellular matrix | Heart Diseases OR Heart Failure

**Appendix 2**

Appendix 2 table 1. List of clinical trial registries and identification number.

| **Clinical trial registry title** | **Trial identification number** |
| --- | --- |
| Efficacy and Safety of Intracoronary Ad5FGF-4 in Patients With Stable Angina (AGENT-3) | NCT00346437 |
| Study to Examine the Safety and Effectiveness of Implanted Skeletal Muscle Cells (Cells Removed From the Thigh Muscle) Into Scarred Areas of Heart Muscle After Heart Attack. | NCT00102128 |
| Bone Marrow Transfer to Enhance ST-Elevation Infarct Regeneration | NCT00224536 |
| Efficacy Study of Intramuscular or Intracoronary Injection of Autologous Bone Marrow Cells to Treat Scarred Myocardium | NCT00560742 |
| Stem Cell Mobilization to Treat Chest Pain and Shortness of Breath in Patients With Coronary Artery Disease | NCT00043628 |
| Cell Therapy for Coronary Heart Disease | NCT00289822 |
| NOGA Angiogenesis Revascularization Therapy: Evaluation by RadioNuclide Imaging - The Northern Trial | NCT00143585 |
| Safety and Efficacy of Intracoronary Ad5FGF-4 in Patients With Stable Angina | NCT00185263 |
| Stem Cells in Myocardial Infarction | NCT00135928 |
| Autologous Stem Cell Transplantation in Acute Myocardial Infarction | NCT00199823 |
| Bone Marrow-Derived Stem Cell Transfer in Acute Myocardial Infarctions | NCT00264316 |
| Long Term Follow-up of Autologous Bone Marrow Mononuclear Cells Therapy in STEMI | NCT00626145 |
| The Effect of Mobilized Stem Cell by G-CSF and VEGF Gene Therapy in Patients With Stable Severe Angina Pectoris | NCT00135850 |
| Bone Marrow Stem Cell Mobilisation Therapy for Acute Myocardial Infarction (AMI)(REVIVAL-2) | NCT00126100 |
| BONAMI (BOne Marrow in Acute Myocardial Infarction) | NCT00200707 |
| Autologous Stem Cells for Cardiac Angiogenesis (FOCUS HF) | NCT00203203 |
| REPAIR-AMI: Intracoronary Progenitor Cells in Acute Myocardial Infarction (AMI) | NCT00279175 |
| Progenitor Cell Therapy in Dilative Cardiomyopathy | NCT00284713 |
| Myocardial Regeneration and Angiogenesis in Myocardial Infarction With G-CSF and Intra-Coronary Stem Cell Infusion-3-DES | NCT00291629 |
| Myocardial Regeneration and Angiogenesis in Myocardial Infarction With G-CSF and Intra-Coronary Stem Cell Infusion | NCT00307879 |
| Stem Cell Therapy to Improve Myocardial Function in Patients With Acute Myocardial Infarction | NCT00316381 |
| CD133+ Autologous Cells After Myocardial Infarction | NCT00400959 |
| Autologous Transplantation of Bone Marrow Mononuclear Stem-Cells by Mini-Thoracotomy | NCT00615394 |
| Stem Cell Study for Patients With Heart Disease | NCT00081913 |
| EPC by Intracoronary Injection in Patients With Chronic Stable Angina | NCT00384514 |
| To Find the Best Dose of pVGI.1(VEGF2) to Benefit Angina Patients When Given With an Experimental Injection Catheter | NCT00090714 |
| Endothelial Modulation for Angiogenic Therapy | NCT00134433 |
| Angiogenesis Using VEGF-A165/bFGF Plasmid Delivered Percutaneously in No-option CAD Patients; a Controlled Trial | NCT00620217 |
| Safety Study of Adult Mesenchymal Stem Cells (MSC) to Treat Acute Myocardial Infarction | NCT00114452 |
| Autologous/Allogeneic Progenitor Stem Cell Therapy for Congestive Heart Failure | NCT00128258 |
| Stem Cell Therapy in Chronic Ischemic Heart Failure | NCT00235417 |
| Bone Marrow Stem Cell Infusion Following a Heart Attack | NCT00268307 |
| Bone Marrow Cells in Myocardial Infarction | NCT00363324 |
| Myocardial Stem Cell Administration After Acute Myocardial Infarction (MYSTAR) Study | NCT00384982 |
| Safety and Efficacy of Bone Marrow Cell Transplantation in Humans Myocardial Infarction | NCT00437710 |
| Autologous Bone Marrow Transplanted Via Transendocardial Catheter to Chronic Myocardial Infarct Border Zone | NCT00507468 |
| Intracoronary Stem Cell Therapy in Patients With Acute Myocardial Infarction (SCAMI) | NCT00669227 |
| Autologous Transplantation of Bone Marrow Mononuclear Stem-Cells for Dilated Cardiomyopathy | NCT00743639 |
| Bone Marrow Derived Adult Stem Cells for Chronic Heart Failure | NCT00747708 |
| Randomized Evaluation of Intracoronary Transplantation of Bone Marrow Stem Cells in Myocardial Infarction | NCT00874354 |
| Stem Cell Study for Patients With Heart Disease | NCT00221182 |
| Stem Cell Therapy for Vasculogenesis in Patients With Severe Myocardial Ischemia | NCT00260338 |
| A Study to Treat Patients Whose Chronic Angina Symptoms Are Not Relieved by Medication and Have an Area of the Heart That Cannot be Treated by Standard Therapies | NCT00215696 |
| Safety and Effects of Implanted (Autologous) Skeletal Myoblasts (MyoCell) Using an Injection Catheter = SEISMIC Trial | NCT00375817 |
| Treatment of Myocardial Infarction With Bone Marrow Derived Stem Cells | NCT00275977 |
| Cell Repair in Heart Failure | NCT00285454 |
| Intra-coronary Infusion of Bone Marrow Derived Autologous CD34+ Selected Cells in Patients With Acute Myocardial Infarction | NCT00313339 |
| Cell-Wave Study: Combined Extracorporal Shock Wave Therapy and Intracoronary Cell Therapy in Chronic Ischemic Myocardium | NCT00326989 |
| Cell Therapy In Dilated Cardiomyopathy | NCT00333827 |
| Cell Therapy in Chagas Cardiomyopathy | NCT00349271 |
| Cell Therapy in Myocardial Infarction | NCT00350766 |
| SWiss Multicenter Intracoronary Stem Cells Study in Acute Myocardial Infarction (SWISS-AMI) | NCT00355186 |
| Cell Therapy in Chronic Ischemic Heart Disease | NCT00362388 |
| Intracoronary Stem Cells in Large Myocardial Infarction | NCT00389545 |
| Combined CABG and Stem-Cell Transplantation for Heart Failure | NCT00418418 |
| ACPs Combined With CABG in Patients With CHF | NCT00523224 |
| Safety and Efficacy Study of Stem Cell Transplantation to Treat Dilated Cardiomyopathy | NCT00629018 |
| ACT34-CMI -- Adult Autologous CD34+ Cells | NCT00300053 |
| Intramyocardial Injection of Autologous Aldehyde Dehydrogenase-Bright Stem Cells for Therapeutic Angiogenesis (FOCUS Br) | NCT00314366 |
| Safety and Efficacy of Autologous, Intracoronary Stem Cell Injections in Total Coronary Artery Occlusions | NCT00365326 |
| Percutaneous Randomised Infusion of Marrow Aspirate To Improve Ventricular Efficiency | ISRCTN74381875 |
| Bone Marrow Cell Transplantation to Improve Heart Function in Individuals With End-Stage Heart Failure | NCT00383630 |
| Randomized Clinical Trial of Adipose-Derived Stem Cells in the Treatment of Pts With ST-elevation Myocardial Infarction | NCT00442806 |
| Congestive Heart Failure Surgical Treatmment With Autologous Stem Cell Therapy | NCT00480961 |
| MAGIC Cell-5-Combicytokine Trial | NCT00501917 |
| To Assess Safety and Efficacy of Myoblast Implantation Into Myocardium Post Myocardial Infarction | NCT00526253 |
| A Trial Using CD133 Enriched Bone Marrow Cells Following Primary Angioplasty for Acute Myocardial Infarction | NCT00529932 |
| Combination Stem Cell (MESENDO) Therapy for Utilization and Rescue of Infarcted Myocardium | NCT00548613 |
| Prospective Randomized Study of Mesenchymal Stem Cell Therapy in Patients Undergoing Cardiac Surgery (PROMETHEUS) | NCT00587990 |
| Endocardial Stem Cells Approach Efficacy | NCT00841958 |
| Study of the Efficacy of Percutaneous Implantation of Autologous Myoblasts in Patients With Old Infarction | NCT00908622 |
| Stem Cell Therapy to Improve Myocardial Function in Patients Undergoing Coronary Artery Bypass Grafting (CABG) | NCT00395811 |
| Vescell(TM) for the Treatment of Patients With Severe Anginal Syndrome With or Without Heart Failure | NCT00416663 |
| A Randomized Clinical Trial of Adipose-derived Stem Cells in Treatment of Non Revascularizable Ischemic Myocardium | NCT00426868 |
| Bypass Surgery and CD133 Marrow Cell Injection for Treatment of Ischemic Heart Failure | NCT00462774 |
| Stem Cell Therapy as Adjunct to Revascularization | NCT00463853 |
| Angiogenesis in Women With Angina Pectoris Who Are Not Candidates for Revascularization | NCT00438867 |
| Efficacy and Safety Study of Genetically Targeted Enzyme Replacement Therapy for Advanced Heart Failure | NCT00454818 |
| Induced Angiogenesis by Genic Therapy in Advanced Ischemic Cardiomyopathy | NCT00744315 |
| Safety Study of Allogeneic Mesenchymal Precursor Cells (MPCs) in Subjects With Recent Acute Myocardial Infarction | NCT00555828 |
| Stem Cell Study for Subjects With Congestive Heart Failure | NCT00620048 |
| Intracoronary Infusion of Autologous Bone Marrow Cells for Treatment of Idiopathic Dilated Cardiomyopathy | NCT00629096 |
| Autologous Mesenchymal Stromal Cell Therapy in Heart Failure | NCT00644410 |
| Use of Adult Autologous Stem Cells in Treating People Who Have Had a Heart Attack (The TIME Study) | NCT00684021 |
| Use of Adult Autologous Stem Cells in Treating People 2 to 3 Weeks After Having a Heart Attack (The Late TIME Study) | NCT00684060 |
| By Pass Surgery With Stem Cell Therapy in Chronic Ischemic Cardiopathy | NCT00690209 |
| Effects of Intracoronary Progenitor Cell Therapy on Coronary Flow Reserve After Acute MI | NCT00711542 |
| A Phase II Dose-escalation Study to Assess the Feasibility and Safety of Transendocardial Delivery of Three Different Doses of Allogeneic Mesenchymal Precursor Cells (MPCs)in Subjects With Heart Failure | NCT00721045 |
| Intracoronary Autologous Stem Cell Transplantation in ST Elevation Myocardial Infarction: TRACIA STUDY. | NCT00725738 |
| Bone Marrow Derived Adult Stem Cells for Acute Anterior Myocardial Infarction | NCT00765453 |
| Use of Ixmyelocel-T (Formerly Cardiac Repair Cell [CRC] Treatment) in Patients With Heart Failure Due to Dilated Cardiomyopathy (IMPACT-DCM) | NCT00765518 |
| The Transendocardial Autologous Cells (hMSC or hBMC) in Ischemic Heart Failure Trial (TAC-HFT) | NCT00768066 |
| Stem Cells and Resynchronization Cardiac | NCT00800657 |
| C-Cure Clinical Trial | NCT00810238 |
| Combination Stem Cell Therapy for the Treatment of Severe Coronary Ischemia | NCT00643981 |
| Safety and Efficacy of Autologous Endothelial Progenitor Cell CD 133 for Therapeutic Angiogenesis | NCT00694642 |
| Phase II Combination Stem Cell Therapy for the Treatment of Severe Coronary Ischemia(CI) | NCT00790764 |
| Stem Cell Mobilization and VEGF Gene Transfer for Heart Failure | NCT00279539 |
| Prochymal® (Human Adult Stem Cells) Intravenous Infusion Following Acute Myocardial Infarction (AMI) | NCT00877903 |
| Cardiac Stem Cell Infusion in Patients With Ischemic CardiOmyopathy (SCIPIO) | NCT00474461 |
| Safety and Efficacy on Cell-based Therapy in Patients With Recent Large Acute Myocardial Infarction | NCT00691834 |
| Effectiveness of Stem Cell Treatment for Adults With Ischemic Cardiomyopathy (The FOCUS Study) | NCT00824005 |
| Ex Vivo Cultured Bone Marrow Derived Allogenic MSCs in AMI | NCT00883727 |
| CArdiosphere-Derived aUtologous Stem CElls to Reverse ventricUlar dySfunction | NCT00893360 |
| Effect of Intramyocardial Injection of Mesenchymal Precursor Cells on Heart Function in People Receiving an LVAD | NCT00927784 |
| Intracoronary Injection of Autologous Bone Marrow Cells in Patients With Chronic Heart Failure: Five Years Follow up | NCT00933621 |
| The Enhanced Angiogenic Cell Therapy - Acute Myocardial Infarction Trial | NCT00936819 |
| Bone Marrow Derived Mononuclear Cells For Myocardial Regeneration | NCT00938847 |
| Alster Stem Cells - Intramyocardial Stem Therapy | NCT00939042 |
| Strengthening Transplantation Effects of Bone Marrow Mononuclear Cells With Atorvastatin in Myocardial Infarction | NCT00979758 |
| AutoLogous Human CArdiac-Derived Stem Cell to Treat Ischemic cArdiomyopathy (ALCADIA) | NCT00981006 |
| Trial of Hematopoietic Stem Cells in Acute Myocardial Infarction | NCT00984178 |
| Use of Ixmyelocel-T (Formerly Catheter-based Cardiac Repair Cell [CRC]) Treatment in Patients With Heart Failure Due to Dilated Cardiomyopathy | NCT01020968 |
| Intramyocardial Transplantation of Bone Marrow Stem Cells in Addition to Coronary Artery Bypass Graft (CABG) Surgery | NCT00950274 |
| IMPACT-CABG Trial: IMPlantation of Autologous CD133+ sTem Cells in Patients Undergoing CABG | NCT01033617 |
| Injection of Autologous Bone Marrow Cells into Damaged Myocardium of No-option Patients with Ischemic Heart Failure: a randomized placebo controlled trail. - Cell therapy for ischemic heart failure | EUCTR2009-016364-36-NL |
| Comparison of intracoronary selected CD 133+ bone marrow stem cells in cardiac recovery after acute myocardial infarct and left ventricular dysfunction: COMPARE-AMI a randomized controlled double blind clinical study | ACTRN12609001045202 |
| EndocardialVascularEndothelialGrowth Factor D(VEGF-D)Gene Therapy for the Treatment of Severe Coronary Heart Disease | NCT01002430 |
| Gene Therapy for the Treatment of Chronic Stable Angina | NCT01002495 |
| A Pilot Study Investigating the Clinical Use of Tissue Engineered Vascular Grafts in Congenital Heart Surgery | NCT01034007 |
| Stem Cell Study for Patients With Heart Failure | NCT00346177 |
| CD133+ Cell Therapy for Refractory Coronary Heart Disease | NCT01049867 |
| Prospective, Controlled and Randomized Clinical Trial on Cardiac Cell Regeneration With Laser and Autologous Bone Marrow Stem Cells, in Patients With Coronary Disease and Refractory Angina | NCT01214499 |
| BMAC Enhanced Coronary Artery Bypass Grafting (CABG) | NCT01061580 |
| Feasibility Study of BMAC Enhanced CABG | NCT01074099 |
| Mesenchymal Stem Cells and Myocardial Ischemia | NCT01076920 |
| Bone Marrow Derived Mononuclear Cells for Ischemic Cardiomyopathy | NCT01144221 |
| Direct Endomyocardial Injection of Autologous Bone Marrow Cells to Treat Ischaemic Heart Failure | NCT01150175 |
| Autologous Bone Marrow Derived Stem Cells for Acute Myocardial Infarction | NCT01167751 |
| Bone Marrow Derived AC 133+ and Mono-Nuclear Cells (MNC) Implantation in Myocardial Infarction (MI) Patient | NCT01187654 |
| Safety and Efficacy of Adipose Derived Regenerative Cells (ADRCs) Delivered Via the Intracoronary Route in the Treatment of Patients With ST-elevation Acute Myocardial Infarction (AMI) | NCT01216995 |
| Intramuscular Injection of Mesenchymal Stem Cell for Treatment of Children With Idiopathic Dilated Cardiomyopathy | NCT01219452 |
| Clinical Study of Hypoxia-Stressed Bone Marrow Mononuclear Cell Transplantation to Treat Heart Diseases | NCT01234181 |
| Cell Therapy in Patients With Chronic Ischemic Heart Disease Undergoing Cardiac Surgery | NCT01267331 |
| AC6 Gene Transfer for CHF | NCT00787059 |
| Study to Evaluate the Safety of a Single Escalating Dose of ACRX-100 in Adults With Ischemic Heart Failure | NCT01082094 |
| Plasmonic Photothermal and Stem Cell Therapy of Atherosclerosis Versus Stenting | NCT01436123 |
| MesenchYmal STROMAL CELL Therapy in Patients With Chronic Myocardial Ischemia (MyStromalCell Trial) | NCT01449032 |
| Stem Cell Therapy in Patients With Severe Heart Failure & Undergoing Left Ventricular Assist Device Placement | NCT00869024 |
| Efficacy assessment of repeat intramyocardial injection of autologous bone marrow cells in previously responding no-option patients with residual refractory angina pectoris and documented ischemia | EUCTR2009-017924-18 |
| Transcoronary Infusion of Cardiac Progenitor Cells in Patients With Single Ventricle Physiology | NCT01273857 |
| Intracoronary Human Wharton's Jelly- Derived Mesenchymal Stem Cells (WJ-MSCs) Transfer in Patients With Acute Myocardial Infarction (AMI) | NCT01291329 |
| Retrograde Delivery of BMAC (Bone Marrow Aspirate Concentrate) for Congestive Heart Failure | NCT01299324 |
| Bone Marrow Derived Adult Stem Cells for Dilated Cardiomyopathy | NCT01302171 |
| Intra-coronary Versus Intramyocardial Application of Enriched CD133pos Autologous Bone Marrow Derived Stem Cells | NCT01337011 |
| Safety and Efficacy Study of Intramyocardial Stem Cell Therapy in Patients With Dilated Cardiomyopathy | NCT01350310 |
| Autologous Cell Therapy for Ischemic Heart Failure | NCT01353690 |
| Intramyocardial Multiple Precision Injection of Bone Marrow Mononuclear Cells in Myocardial Ischemia | NCT01354678 |
| Safety and Efficacy of Intracoronary Adult Human Mesenchymal Stem Cells After Acute Myocardial Infarction | NCT01392105 |
| PercutaneOus StEm Cell Injection Delivery Effects On Neomyogenesis in Dilated CardioMyopathy (The POSEIDON-DCM Study) | NCT01392625 |
| ESTIMATION Study for Endocardial Mesenchymal Stem Cells Implantation in Patients After Acute Myocardial Infarction | NCT01394432 |
| The Effect of Intramyocardial Injection of Mesenchymal Precursor Cells on Myocardial Function in Patients Undergoing LVAD Implantation | NCT01442129 |
| Safety and Feasibility Study of Umbilical Cord Blood Cells for Infants With Hypoplastic Left Heart Syndrome | NCT01445041 |
| Intracoronary Infusion of Bone Marrow Mononuclear Cells in Patients With Previous Myocardial Infarction. | NCT01454323 |
| Allogeneic Heart Stem Cells to Achieve Myocardial Regeneration | NCT01458405 |
| IMPACT-CABG Trial: IMPlantation of Autologous CD133+ sTem Cells in Patients Undergoing Coronary Artery Bypass Grafting | NCT01467232 |
| NBS10 (Also Known as AMR-001) Versus Placebo Post ST Segment Elevation Myocardial Infarction | NCT01495364 |
| REgenerative CardiOsphere iNjection to STRengthen dysfUnCTional Hearts | NCT01496209 |
| Safety and Efficacy of Adipose Derived Stem Cells for Non-Ischemic Congestive Heart Failure | NCT01502501 |
| Safety and Efficacy of Adipose Derived Stem Cells for Congestive Heart Failure | NCT01502514 |
| Intra-coronary freshly isolated bone marrow cells transplantation improve cardiac function in patients with ischemic heart disease | ISRCTN54510226 |
| Safety Study of Gene Therapy for Ischemic Heart Disease in Korea | NCT01422772 |
| A Randomized, Controlled Study to Evaluate Algisyl-LVR™ as a Method of Left Ventricular Augmentation for Heart Failure | NCT01311791 |
| Efficacy and Safety of Targeted Intramyocardial Delivery of Auto CD34+ Stem Cells for Improving Exercise Capacity in Subjects With Refractory Angina | NCT01508910 |
| Stem Cell Injection to Treat Heart Damage During Open Heart Surgery | NCT01557543 |
| Intracardiac CD133+ Cells in Patients With No-option Resistant Angina | NCT01660581 |
| Stem Cell Implantation in Patients Undergoing CABG | NCT01721902 |
| Autologous Stem Cells in Pediatric Patients With Dilated Cardiomyopathy | NCT01504594 |
| Rapid Delivery of Autologous Bone Marrow Derived Stem Cells in Acute Myocardial Infarction Patients. | NCT01536106 |
| Safety and Feasibility Trial of Adipose-Derived Regenerative Cells in the Treatment of Chronic Myocardial Ischemia | NCT01556022 |
| BAMI. The Effect of Intracoronary Reinfusion of Bone Marrow-derived Mononuclear Cells(BM-MNC) on All Cause Mortality in Acute Myocardial Infarction | NCT01569178 |
| Implantation of Peripheral Stem Cells in Patient With Ischemic Cardiomyopathy | NCT01615250 |
| Stem Cell Therapy in Patients With Myocardial Infarction and Persistent Total Occlusion of Infarct Related Artery | NCT01625949 |
| To Evaluate the Efficacy and Safety of Hearticelgram®-AMI in Patients With Acute Myocardial Infarction. | NCT01652209 |
| METHOD - Bone Marrow Derived Mononuclear Cells in Chronic Ischemic Disease | NCT01666132 |
| An Efficacy, Safety and Tolerability Study of Ixmyelocel-T Administered Via Transendocardial Catheter-based Injections to Subjects With Heart Failure Due to Ischemic Dilated Cardiomyopathy (IDCM) | NCT01670981 |
| Compare the Effects of Single Versus Repeated Intracoronary Application of Autologous Bone Marrow-derived Mononuclear Cells on Mortality in Patients With Chronic Post-infarction Heart Failure | NCT01693042 |
| Clinical Trial of Autologous Adipose Tissue Derived Stromal Cell Therapy for Ischemic Heart Failure | NCT01709279 |
| Intracoronary Autologous Mesenchymal Stem Cells Implantation in Patients With Ischemic Dilated Cardiomyopathy | NCT01720888 |
| Cell Therapy in Severe Chronic Ischemic Heart Disease | NCT01727063 |
| Randomized Clinical Trial of Intravenous Infusion Umbilical Cord Mesenchymal Stem Cells on Cardiopathy | NCT01739777 |
| The Long-term and Short-term Efficacy and Safety of Transplantation Autologous Bone Marrow Cells (BMCs) in Patients With the First STEMI (ST Segment Elevation Myocardial Infarction) | NCT01748383 |
| Allogeneic Stem Cells Implantation Combined With Coronary Bypass Grafting in Patients With Ischemic Cardiomyopathy | NCT01753440 |
| Efficacy and Safety of Bone Marrow-Derived Mesenchymal Cardiopoietic Cells (C3BS-CQR-1) for the Treatment of Chronic Advanced Ischemic Heart Failure | EUCTR2011-001117-13 |
| A Study of Genetically Targeted Enzyme Replacement Therapy for Advanced Heart Failure | NCT01643330 |
| Efficacy and Safety of Ad5FGF-4 for Myocardial Ischemia in Patients With Stable Angina Due to Coronary Artery Disease | NCT01550614 |
| Administration of AdVEGF-All6A+ to Myocardium of Individuals With Diffuse CAD Via Minimally Invasive Surgery | NCT01757223 |
| Study Protocol of Intramyocardial Injection of Autologous Bone Marrow Stem Cells for Refractory Angina | NCT01966042 |
| Transplantation of Autologous Cardiac Stem Cells in Ischemic Heart Failure | NCT01758406 |
| Left Ventricular Assist Device Combined With Allogeneic Mesenchymal Stem Cells Implantation in Patients With End-stage Heart Failure. | NCT01759212 |
| Safety and Efficacy of Autologous Cardiopoietic Cells for Treatment of Ischemic Heart Failure. | NCT01768702 |
| A Study of Allogeneic Mesenchymal Bone Marrow Cells in Subjects With ST Segment Elevation Myocardial Infarction (STEMI) | NCT01770613 |
| Safety Study of Allogeneic Mesenchymal Precursor Cell Infusion in Myocardial Infarction | NCT01781390 |
| Cardiac Progenitor Cell Infusion to Treat Univentricular Heart Disease (PERSEUS) | NCT01829750 |
| Safety Study of Autologous Umbilical Cord Blood Cells for Treatment of Hypoplastic Left Heart Syndrome | NCT01883076 |
| Mesenchymal Stem Cells to Treat Ischemic Cardiomyopathy | NCT01913886 |
| Umbilical Cord Derived Mesenchymal Stem Cells Therapy in Ischemic Cardiomyopathy | NCT01946048 |
| STem cElls Mobilization in Acute Myocardial Infarction Outcome Trial | NCT01969890 |
| Study to Assess the Safety and Cardiovascular Effects of Autologous Adipose-Derived Stromal Cells Implantation In Patients During the Acute Recovery Phase of ST-Elevation Myocardial Infarction | NCT01974128 |
| Intracoronary Infusion of Mononuclear Cells Autologous Bone Marrow in Patients With Chronic Coronary Occlusion and Ventricular Dysfunction, Previously Revascularized. | NCT02022514 |
| AAV1-CMV-Serca2a GENe Therapy Trial in Heart Failure | NCT01966887 |
| The TRansendocardial Stem Cell Injection Delivery Effects on Neomyogenesis STudy (The TRIDENT Study) | NCT02013674 |
| Autologous Bone Marrow Mononuclear Cells in the Combined Treatment of Coronary Heart Disease | NCT02059512 |
| Efficacy and Safety of Allogeneic Mesenchymal Precursor Cells (Rexlemestrocel-L) for the Treatment of Heart Failure. | NCT02032004 |
| Infusion Intracoronary of Mononuclear Autologous Adult no Expanded Stem Cells of Bone Marrow on Functional Recovery in Patients With Idiopathic Dilated Cardiomyopathy and Heart Failure. | NCT02033278 |
| Safety & Efficacy of Adipose-Derived Regenerative Cells in the Treatment of Chronic Myocardial Ischemia (ATHENA II) | NCT02052427 |
| Transplantation of Human Embryonic Stem Cell-derived Progenitors in Severe Heart Failure | NCT02057900 |
| Phase I Trial of Endocavitary Injection of Bone Marrow Derived CD133+ Cells in Ischemic Refractory Cardiomyopathy (RECARDIO Trial) | NCT02059681 |
| Repetitive Intramyocardial CD34+ Cell Therapy in Dilated Cardiomyopathy (REMEDIUM) | NCT02248532 |
| Intracoronary Transplantation of Bone Marrow Derived Mononuclear Cells in Pediatric Cardiomyopathy | NCT02256501 |
| A Phase 2 Trial of AMI MultiStem® Therapy in Subjects With Non-ST Elevation Acute Myocardial Infarction | NCT02277613 |
| Dilated cardiomYopathy iNtervention With Allogeneic MyocardIally-regenerative Cells (DYNAMIC) | NCT02293603 |
| Congestive Heart Failure Cardiopoietic Regenerative Therapy (CHART-2) Trial - THE CHART-2 TRIAL | NCT02317458 |
| Human Umbilical Cord Stroma MSC in Myocardial Infarction | NCT02323477 |
| Impact of Intracoronary Injection of Autologous BMMC for LV Contractility and Remodeling in Patients With STEMI | NCT02323620 |
| Investigation of the Safety and Feasibility of AAV1/SERCA2a Gene Transfer in Patients With Chronic Heart Failure | NCT00534703 |
| Novel Regenerative Therapy with Autologus Myoblast Sheet for Pediatric Heart Failure patients | JPRN-UMIN000013941 |
| Mesenchymal Stem Cell Administration in the Treatment of Coronary Graft Disease in Heart Transplant Patients | NCT02472002 |
| Safety & Efficacy of Intramyocardial Injection of Mesenchymal Precursor Cells on Myocardial Function in LVAD Recipients | NCT02362646 |
| Intracoronary or Intravenous Infusion Human Wharton' Jelly-derived Mesenchymal Stem Cells in Patients With Ischemic Cardiomyopathy | NCT02368587 |
| CSCC_ASC Therapy in Patients With Severe Heart Failure | NCT02387723 |
| Allogeneic hMSC Injection in Patients With Hypoplastic Left Heart Syndrome | NCT02398604 |
| Bone Marrow Derived Mesenchymal Stem Cells in Improving Heart Function in Patients With Heart Failure Caused by Anthracyclines | NCT02408432 |
| Timing for Bone Marrow Mononuclear Cells After Acute Myocardial Infarction | NCT02425358 |
| CardiAMP™ Cell Therapy Heart Failure Trial | NCT02438306 |
| Safety and Efficacy Evaluation of Intracoronary Infusion of Allogeneic Human Cardiac Stem Cells in Patients With AMI | NCT02439398 |
| Human Umbilical-Cord-Derived Mesenchymal Stem Cell Therapy in Ischemic Cardiomyopathy | NCT02439541 |
| Bone Marrow-derived Mesenchymal Stem Cells (MSC) Administration in Weaning From Left Ventricular Assist Device | NCT02460770 |
| Administration of Mesenchymal Stem Cells in Patients With Chronic Ischemic Cardiomyopathy (MESAMI2) | NCT02462330 |
| A Study to Assess the Effect of Intravenous Dose of (aMBMC) to Subjects With Non-ischemic Heart Failure | NCT02467387 |
| A Trial of Autologous Bone Marrow Derived Stem Cells in Paediatric Heart Failure | NCT02479776 |
| Combination of Mesenchymal and C-kit+ Cardiac Stem Cells as Regenerative Therapy for Heart Failure | NCT02501811 |
| The Transendocardial Autologous Cells (hMSC) or (hMSC) and (hCSC) in Ischemic Heart Failure Trial. | NCT02503280 |
| Therapy of Preconditioned Autologous BMMSCs for Patients With Ischemic Heart Disease | NCT02504437 |
| Phase I Safety and Feasibility Study of Intracoronary Delivery of Autologous Bone Marrow Derived Mononuclear Cells | NCT02549625 |
| A Phase 1/2 Study of High-dose Genetically Targeted Enzyme Replacement Therapy for Advanced Heart Failure | NCT02346422 |
| Human Umbilical Cord-derived Mesenchymal Stem Cells With Injectable Collagen Scaffold Transplantation for Chronic Ischemic Cardiomyopathy | NCT02635464 |
| Clinical trial on the safety of myocardium regenerative therapy for ischemic heart disease using biodegradable polymeric carrier and basic fibroblast growth factor | JPRN-UMIN000018709 |
| Safety and Exploratory Efficacy Study of UCMSCs in Patients With Ischemic Heart Disease (SEESUPIHD) | NCT02666391 |
| EXCELLENT (EXpanded CELL ENdocardiac Transplantation) | NCT02669810 |
| A Study of Allogeneic Low Oxygen Mesenchymal Bone Marrow Cells in Subjects With Myocardial Infarction | NCT02672267 |
| Stem Cell Therapy in IschEmic Non-treatable Cardiac Disease | NCT02673164 |
| Cardiac Stem/Progenitor Cell Infusion in Univentricular Physiology (APOLLON Trial) | NCT02781922 |
| Stem Cells Therapy in Advanced Heart Failure | NCT02871466 |
| Study of Autologous Bone Marrow Derived Mononuclear Cells for Treatment of Ebstein Anomaly | NCT02914171 |
| Cell Therapy in HFpEF | NCT02923609 |
| Regression of Fibrosis & Reversal of Diastolic Dysfunction in HFPEF Patients Treated With Allogeneic CDCs | NCT02941705 |
| Donor Bone Marrow Derived Mesenchymal Stem Cells in Controlling Heart Failure in Patients With Cardiomyopathy Caused by Anthracyclines | NCT02962661 |
| Ad-HGF Treatment for Myocardial Infarction | NCT02844283 |
| Autologous Atrial Appendage Derived Cells in the Treatment of Heart Failure | NCT02672163 |
| Stem Cell Heart Injections During Laser Revascularization Surgery for Treatment of Chronic Ischemic Heart Disease | NCT03043742 |
| Transplantation Efficacy of Autologous Bone Marrow Mesenchymal Stem Cells With Intensive Atorvastatin in AMI Patients | NCT03047772 |
| Mesoblast Stem Cell Therapy for Patients With Single Ventricle and Borderline Left Ventricle | NCT03079401 |
| Allogeneic Stem Cell Therapy in Heart Failure | NCT03092284 |
| Transcoronary Infusion of Cardiac Progenitor Cells in Pediatric Dilated Cardiomyopathy | NCT03129568 |
| ICMNC-HF. IntraCoronary Bone Marrow MonoNuclear Cells in Heart Failure (HF) Patients | NCT03145402 |
| The Study of Heart Failure With Human Umbilical Cord Mesenchymal Stem Cells (19#iSCLife®-HF) | NCT03180450 |
| IMMNC-HF: IntraMyocardial Injection of Bone Marrow MonoNuclear Cells in Heart Failure (HF) Patients | NCT03227198 |
| Study to Assess Safety & Effects of Autologous ADSCs During Recovery Phase of ST-Elevation MI Effects of Autologous ADSC Implantation During Subacute Recovery Phase of ST-Elevation Myocardial Infarction | NCT03272191 |
| Japanese OMI Treatment With Kit-positive Cells for Enhanced Regeneration | NCT03351400 |
| Retrograde Application of Bone Marrow Aspirate Concentrate | NCT03372954 |
| Adenovirus Vascular Endothelial Growth Factor D (AdvVEGF-D) Therapy for Treatment of Refractory Angina Pectoris | NCT03039751 |
| AC6 Gene Transfer in Patients With Reduced Left Ventricular Ejection Fraction Heart Failure | NCT03360448 |
| A Pivotal Trial to Establish the Efficacy and Safety of Algisyl in Patients With Moderate to Severe Heart Failure | NCT03082508 |
| Study of Intracoronary CD34+ Cell Administration in Patients With Early Coronary Atherosclerosis | NCT03471611 |
| Safety and Potential Bioactivity of CLBS16 in Patients With Coronary Microvascular Dysfunction and Without Obstructive Coronary Artery Disease | NCT03508609 |
| A Phase IIB Study of the Efficacy and Safety of Intramyocardial Injection of Allogeneic Human Immunomodulatory Progenitor (iMP) cells in Patients Undergoing Coronary Artery Bypass Graft (CABG) Surgery. | NCT03515291 |
| Sequential Treatment of Extra-Corporeal Shock Wave Combined With aUtologous Bone marRow Mesenchymal Stem Cells on Patients With ischEmic Heart Disease : the S-CURE Study | NCT03397095 |
| Cardiovascular Clinical Project to Evaluate the Regenerative Capacity of CardioCell in Patients With Acute Myocardial Infarction (AMI) | NCT03404063 |
| The CHILD Trial: Hypoplastic Left Heart Syndrome Study. | NCT03406884 |
| Randomized Clinical Trial to Evaluate the Regenerative Capacity of CardioCell in Patients With Chronic Ischaemic Heart Failure (CIHF) | NCT03418233 |
| Safety of Autologous Cord Blood Cells in HLHS Patients During Norwood Heart Surgery | NCT03431480 |
| CardiAMP Cell Therapy Chronic Myocardial Ischemia Trial | NCT03455725 |
| Lomecel-B Delivered During Stage II Surgery for Hypoplastic Left Heart Syndrome (ELPIS) | NCT03525418 |
| Intravenous MSC Therapy on Ischemia-Reperfusion Injury in Patients With Myocardial Infarction | NCT03533153 |
| In Vivo Tracking of USPIO Labeled MSC in the Heart | NCT03651791 |
| IPS Differentiated Cardiomyocytes Vein Transplantation for Chronic Heart Failure | NCT03759405 |
| Treating Heart Failure With hPSC-CMs | NCT03763136 |
| Intramyocardial Injection of Autologous Umbilical Cord Blood Derived Mononuclear Cells During Surgical Repair of Hypoplastic Left Heart Syndrome | NCT03779711 |
| Safety and Efficacy Study of Gene Therapy for Acute Myocardial Infarction in Korea | NCT03404024 |
| Novel INXN-4001 Triple Effector Plasmid in Heart Failure | NCT03409627 |
| Use of Bone Marrow Derived Stem Cell and G-CSF With Circulatory Assistance in the Treatment of DCM | NCT03572660 |
| First in Humans to Evaluate Collagen Patches With Stem Cells in Patients With Ischemic Left Ventricular Dysfunction | NCT03746938 |
| Adipose Stromal Cells Injection in the Myocardium for Induction of Revascularization | NCT04005989 |
| MCRcI® Stem Cell Treatment for Diffuse Coronary Artery Disease | NCT04052191 |
| Stem Cell Therapy in Non-IschEmic Non-treatable Dilated CardiomyopathiEs II: a Pilot Study | NCT03797092 |
| UC-MSC Transplantation for Left Ventricular Dysfunction After AMI | NCT03902067 |
| Serial Infusions of Allogeneic Mesenchymal Stem Cells in Cardiomyopathy Patients With Left Ventricular Assist Device | NCT03925324 |
| MiSaver® Stem Cell Treatment for Heart Attack (Acute Myocardial Infarction) | NCT04050163 |
| Evaluate the Safety and Explore Efficacy of Umbilical Cord Mesenchymal Stem Cells in Acute Myocardial Infarction | NCT04056819 |
| Safety of autologous CardAP-cells in patients with dilatative cardiomyopathy: an open, monocentric, double-arm, 3+3 dose escalation, phase I/II study | EUCTR2014-003457-34 |
| Allogeneic adipose derived stromal cell therapy in patients with ischemic heart failure | EUCTR2019-002511-26-DK |
| Gene Therapy for Male Patients With Danon Disease (DD) Using RP-A501; AAV9.LAMP2B | NCT03882437 |
| Epicardial Delivery of XC001 Gene Therapy for Refractory Angina Coronary Treatment (The EXACT Trial) | NCT04125732 |
| Pericardial Matrix With Mesenchymal Stem Cells for the Treatment of Patients With Infarcted Myocardial Tissue | NCT03798353 |
| Randomized Study of Coronary Revascularization Surgery With Injection of WJ-MSCs and Placement of an Epicardial Extracellular Matrix | NCT04011059 |
| WJMSCs Anti-inflammatory Therapy in Coronary Artery Disease | NCT04551456 |
| A Placebo-Controlled Trial of CLBS16 in Subjects With Coronary Microvascular Dysfunction | NCT04614467 |
| Mesenchymal Stromal Cells for Infants With Congenital Heart Disease (MedCaP) | NCT04236479 |
| The Application of the Umbilical Cord Mesenchymal Stem Cells in the Complex Treatment of Chronic Heart Failure of Non-ischemic Etiology | NCT04325594 |
| Stem Cell in Acute Myocardial Infarction | NCT04340609 |
| Bone Marrow Mesenchymal Stem Cells Transfer in Patients With ST-segment Elevation Myocardial Infarction | NCT04421274 |
| Transendocardial Injection of Allogeneic-MSC in Patients With Non-Ischemic Dilated Cardiomyopathy | NCT04476901 |
| WJMSCs Anti-inflammatory Therapy in Acute Myocardial Infarction | NCT04551443 |
| Safety study of induced pluripoteint stem cell-derived cardiac spheres transplantation | JPRN-jRCTa032200189 |
| Heart regeneration therapy by autologous Adipose Tissue Derived Stromal Cells | JPRN-jRCTb040190115 |
| Study to Evaluate Safety, Tolerability, Pharmacokinetics, and Pharmacodynamics of NTLA-2001 in Patients With Hereditary Transthyretin Amyloidosis With Polyneuropathy (ATTRv-PN) and Patients With Transthyretin Amyloidosis-Related Cardiomyopathy (ATTR-CM) | NCT04601051 |
| Safety and Efficacy of Induced Pluripotent Stem Cell-derived Engineered Human Myocardium as Biological Ventricular Assist Tissue in Terminal Heart Failure | NCT04396899 |
| Allogeneic Mesenchymal Human Stem Cell Infusion Therapy for Endothelial DySfunctiOn in Diabetic Subjects With Symptomatic Ischemic Heart Disease. (ACESO-IHD) | NCT04776239 |
| Clinical Trial of Human (Allogeneic) iPS Cell-derived Cardiomyocytes Sheet for Ischemic Cardiomyopathy | NCT04696328 |
| New Horizons for the Treatment of Cardiomyopathy in Children | NCT04893629 |
| Intramyocardial Injection of Autologous UCB-MNC During Fontan Surgery for SRV Dependent CHD | NCT04907526 |
| Evaluation of Lomecel-B™ Injection in Patients With Hypoplastic Left Heart Syndrome: A Phase IIb Clinical Trial. | NCT04925024 |
| Treatment of Heart Failure Using Human Umbilical Cord Mesenchymal Stem Cells(hUC-MSC) | NCT04939077 |
| Treating Congestive HF With hiPSC-CMs Through Endocardial Injection | NCT04982081 |
| Multi-intravenous Infusion of Umbilical Cord Mesenchymal Stem Cells in Heart Failure With Reduced Ejection Fraction（PRIME-HFrEF Study） | NCT04992832 |
| MSCs for Prevention of MI-induced HF | NCT05043610 |
| Human Embryonic Stem Cell-Derived Cardiomyocyte Therapy for Chronic Ischemic Left Ventricular Dysfunction | NCT05068674 |
| Safety of Cultured Allogeneic Adult Umbilical Cord Derived Mesenchymal Stem Cells for Heart Disease | NCT05147766 |
| Calcium Up-Regulation by Percutaneous Administration of Gene Therapy In Cardiac Disease | NCT04703842 |
| ADR-002K for Patients With Ischemic Cardiomyopathy Who Undergo Coronary Artery Bypass Surgery | NCT04695522 |
| Heart Patch for Myocardial Infarction COVID-19 | NCT04728906 |
| Treating Heart Failure With hiPSC-CMs | NCT05223894 |
| AMI-DC in Patients With Anterior Wall Myocardial Infarction | NCT05554484 |
| Allogeneic iPSC-derived Cardiomyocyte Therapy in Patients With Worsening Ischemic Heart Failure | NCT05566600 |
| Gene Therapy for Post-Operative Atrial Fibrillation | NCT05223725 |
| Phase IA Study of AAVrh.10hFXN Gene Therapy for the Cardiomyopathy of Friedreich's Ataxia | NCT05302271 |
| Gene Therapy for Cardiomyopathy Associated With Friedreich's Ataxia | NCT05445323 |
| A Phase 2, Adaptive, Double-blinded, Placebo Controlled, Randomized, Multicenter Trial to Evaluate the Efficacy, Safety and Tolerability of Intracoronary Infusion of NAN-101 in Adult Subjects With New York Heart Association (NYHA) Class III Heart Failure and Non-ischemic Cardiomyopathy | NCT05598333 |
| A Study of VentriGel in Post-MI Patients | NCT02305602 |
| Epicardial Infarct Repair Using CorMatrix®-ECM: Clinical Feasibility Study | NCT02887768 |
| First in Man Study of Implantable Alginate Hydrogel | NCT04781660 |
| Atrial Appendage Micrograft Transplants to Assist Heart Repair After Cardiac Surgery | NCT05632432 |
| Cell Therapy and Myocardial Recovery in Heart Failure Patients Undergoing Left Ventricular Assist Device Support | NCT06154044 |
| Phosphatase Inhibition by Intracoronary Gene Therapy in Subjects With Non-Ischemic NYHA Class III Heart Failure | NCT05598333 |
| Modulation of SERCA2a of Intra-myocytic Calcium Trafficking in Heart Failure With Preserved Ejection Fraction | NCT06061549 |
| Clinical Trial of Human Allogenic Culture-expanded Bone Marrow-derived Mesenchymal Stem Cells (CardiALLO) | NCT05925608 |
| Treatment of Non-ischemic Cardiomyopathies by Intravenous Extracellular Vesicles of Cardiovascular Progenitor Cells | NCT05774509 |
| A Phase 1, Dose Escalation Trial of RP-A601 in Subjects With PKP2 Variant-Mediated Arrhythmogenic Cardiomyopathy (PKP2-ACM) | NCT05885412 |
| Evaluate the Efficacy and Safety of Allogeneic Umbilical Cord Mesenchymal Stem Cells as an Add-On Treatment for Acute ST-elevation Myocardial Infarction (STEMI) Patients | NCT06147986 |
| Autologous Induced Pluripotent Stem Cells of Cardiac Lineage for Congenital Heart Disease | NCT05647213 |
| Study of Safety and Tolerability of TN-201 in Adults With Symptomatic MYBPC3 Mutation-associated HCM | NCT05836259 |
| Co-transplantation of Mesenchymal Stem Cell Derived Exosomes and Autologous Mitochondria for Patients Candidate for CABG Surgery | NCT05669144 |
